# Models of Purkinje cell dendritic tree selection during early cerebellar development

**DOI:** 10.1371/journal.pcbi.1011320

**Published:** 2023-07-24

**Authors:** Mizuki Kato, Erik De Schutter

**Affiliations:** 1 Computational Neuroscience Unit, Okinawa Institute of Science and Technology Graduate University, Tancha, Okinawa, Japan; 2 Department and Graduate Institute of Pharmacology, National Taiwan University College of Medicine, Taipei City, Taiwan; Ernst-Strungmann-Institut, GERMANY

## Abstract

We investigate the relationship between primary dendrite selection of Purkinje cells and migration of their presynaptic partner granule cells during early cerebellar development. During postnatal development, each Purkinje cell grows more than three dendritic trees, from which a primary tree is selected for development, whereas the others completely retract. Experimental studies suggest that this selection process is coordinated by physical and synaptic interactions with granule cells, which undergo a massive migration at the same time. However, technical limitations hinder continuous experimental observation of multiple cell populations. To explore possible mechanisms underlying this selection process, we constructed a computational model using a new computational framework, NeuroDevSim. The study presents the first computational model that simultaneously simulates Purkinje cell growth and the dynamics of granule cell migrations during the first two postnatal weeks, allowing exploration of the role of physical and synaptic interactions upon dendritic selection. The model suggests that interaction with parallel fibers is important to establish the distinct planar morphology of Purkinje cell dendrites. Specific rules to select which dendritic trees to keep or retract result in larger winner trees with more synaptic contacts than using random selection. A rule based on afferent synaptic activity was less effective than rules based on dendritic size or numbers of synapses.

## Author summary

The mature structure of a Purkinje cell, the main neuron of the cerebellum, is composed of a large flat dendritic tree composed of a single or sometimes two primary dendrites. However, this neuron has multiple similar trees during development, and retracts most of them and keeps its primary tree. At the same time the most numerous neuron type in the brain, cerebellar granule cells, migrate from their place of birth on top of Purkinje cells to a final location below. We focused on interactions of the axons of these granule cells with growing Purkinje dendrites as criteria to select the surviving dendritic tree, hypothesizing that developing an efficient network with other neurons is a main goal of neuronal morphology. We constructed and used computational models to investigate detailed physical interactions and communications between Purkinje and granule cells that are difficult to study experimentally during early postnatal stages.

## Introduction

**Fig 1:**
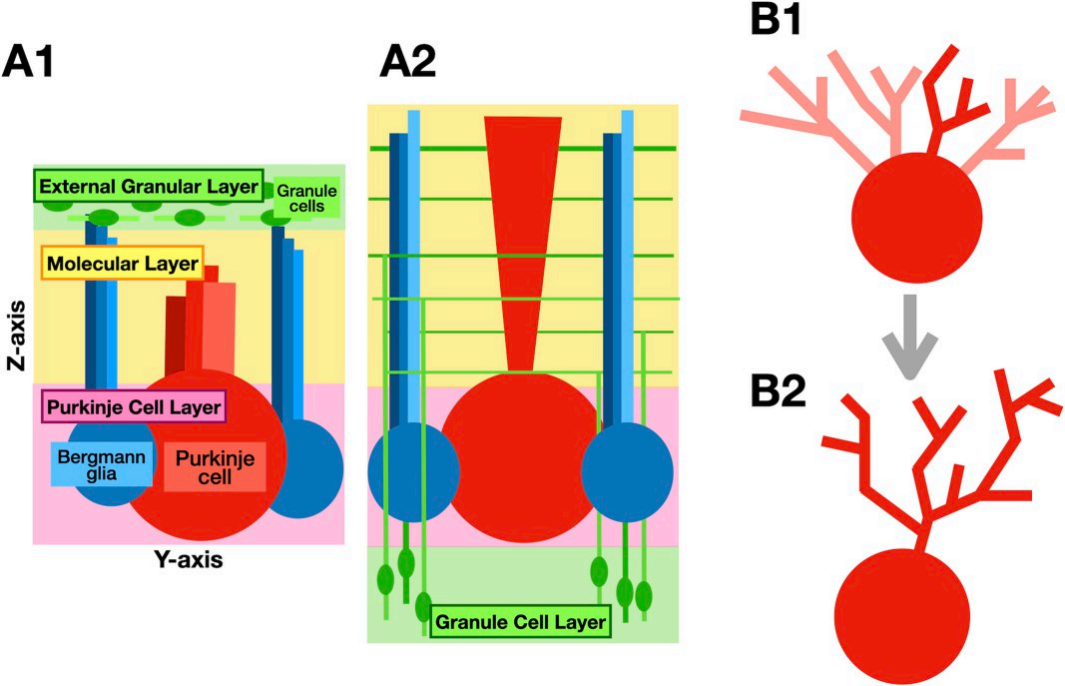
Schematics for dendritic selections of a Purkinje cell and cerebellar layer reconstruction by granule cells. Only main neurons are shown, local inhibitory neurons are ignored. (A1) The cerebellar cortex around postnatal day (P) 1, having granule cell precursors (green oval with light green lines as their leading processes or parallel fibers in the external granular cell layer (shaded green). The middle sheet is the molecular layer (shaded in yellow), and the bottom is the Purkinje cell layer (shaded pink) with cell bodies of the Purkinje cell (red) and Bergmann glia (blue). (A2) Structure of the cerebellar cortex after about P15. All granule cells (green ovals) migrated down from the surface to the bottom to form the granule cell layer (shaded in green). The molecular layer (shaded in yellow) is now at the surface with greatly expanded volume and is filled with Purkinje cell dendrites (red), parallel fibers and descending axons (green) left behind by granule cells. (B1) A Purkinje cell before dendritic selection phase at P4 to P10. The red sphere represents its soma, and initial dendritic trees are light or dark red. (B2) Same Purkinje cell after the selection phase at P8 to P10. In this schematic, the dark red dendritic tree was chosen as its primary tree and other candidate trees were retracted.

The cerebellum is involved in coordinating motor functions as well as in cognition and emotion [1–5]. The cerebellar volume comprises only 10% of the whole brain but holds more than 70% of all neurons. Most of these neurons are cerebellar granule cells whose population seems too numerous to be counted accurately (estimated densities range from 500,000 [6] to 6,562,500 [7] cells per mm^3^ in mice, compared in [8]). The adult mammalian cerebellum consists of a cortex and nuclei, with the cortex containing three layers. A middle layer with cell bodies of its output neuron, the Purkinje cell, an outer molecular layer with Purkinje cell dendrites and granule cell axons and an inner granule cell layer with granule cell somata (Fig 1A2). But at birth cerebellar cortex is largely undeveloped, with a different layering structure. Granule cells proliferate in an external granular layer (Fig 1A1), most of them postnatally, and then migrate through a rapidly expanding molecular layer to arrive in the granular layer. This migration is guided by radial fibers of a specialized glial cell, the Bergmann glia [9–13]. The granule cell axons, parallel fibers and descending axons, are formed during the migration through the molecular layer and establish synaptic contacts with the rapidly growing Purkinje cell dendrites. Adult Purkinje cells have a uniquely flat dendrite, which is optimal to maximize connectivity with perpendicular parallel fibers [14–17], with usually a single root segment. But during early postnatal development, each Purkinje cell grows multiple dendritic trees (defined as the arbor connected to a dendritic root) (Fig 1B1) and then selects a primary tree among them by retracting most of the newly grown dendrites (Fig 1 B2) [18–22]. This is followed by additional growth and development of a more monoplanar tree in the third postnatal week [23].

In isolated *in vitro* environments, Purkinje cells do not fully retract excessive dendritic trees, resulting in persistent multiple primary dendrites [21, 24]. Granule cells and their parallel fibers are strong environmental factors to regulate the dendritic arborizations and retractions in terms of physical and synaptic interactions with Purkinje cells [25–28]. However, the mechanisms by which granule cells select the final primary dendritic tree remains unclear.

Because it is challenging to investigate the dendritic selection stage of Purkinje cells *in vivo* using time-lapse imaging [29], resulting in very small data sets, we decided on a computational approach. Our primary goal is to investigate the interaction between the massive granule cell migration and growth and retraction of Purkinje cell dendritic trees, and to compare different hypothetical selection rules for selection of the winner dendritic tree.

We use the NeuroDevSim software [30], a parallelized agent-based simulation environment to model neural development. NeuroDevSim simulates development as consecutive cycles, in this study we simulate from P1 to P14 and a cycle corresponds roughly to 2.2 hours of growth. Neural growth always starts with a soma, that can be ‘born’ at any cycle and is either stationary (Purkinje cells, Bergmann glia) or migrates (granule cells). Growth of dendrites and axons is represented by cylindrical ‘fronts’ that act as independent agents, fronts are usually active during one cycle only. As is standard for neural growth algorithms [14, 31–33], new fronts can at the next cycle form one (extension) or two (branching) new fronts, that will become their children in the tree-like structure, or they can terminate growth. The choice is determined by neuron specific growth rules that compute probabilities for extension, branching or termination and by the local environment (see Materials and methods). An important effect of the local environment is crowding, fronts cannot overlap with other fronts of the same or different neurons. In addition, attraction and repulsion is also used for specific structures.

Using this computational approach, we first build a model of granule cell migration along Bergmann glia processes and of parallel fiber development that will provide an environment in which Purkinje dendrites can grow. Next, we introduce growth of multiple Purkinje cell dendrites starting at P4. Before we compare possible selection mechanisms, we investigate control scenarios with random selection, no selection, or no environment.

## Results

### Granule cell migration model

**Fig 2:**
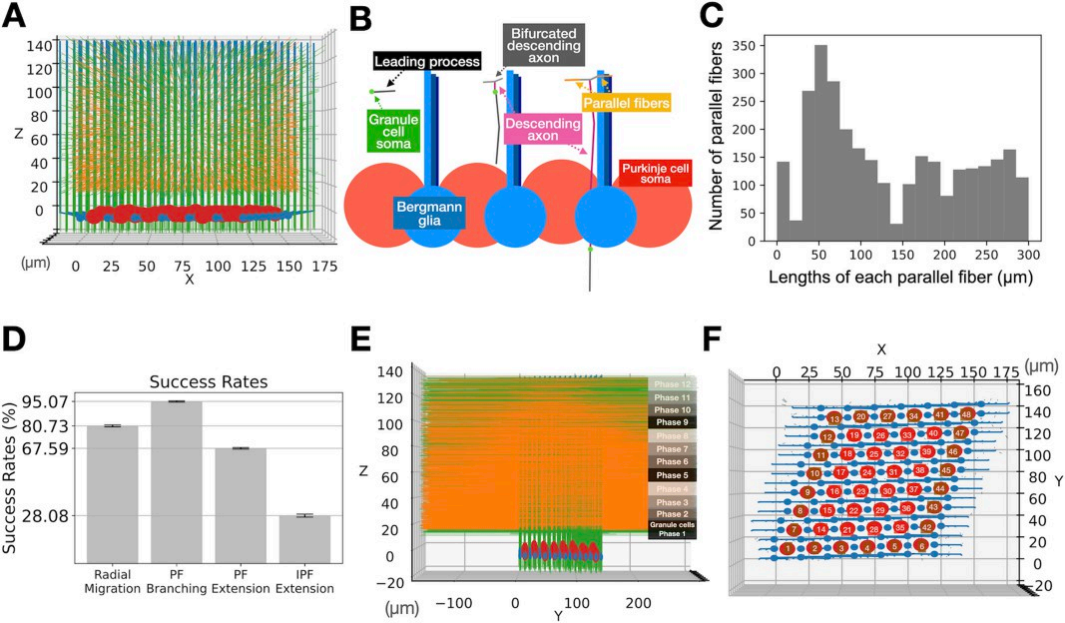
Granule cell migration model as an environment for Purkinje cell growth. (A) A representative simulation cube at the end of the simulation visualized from X side. Red large spheres are Purkinje cell somas. Blue small spheres with processes growing upward are Bergmann glia. Numerous green thin threads are axons of granule cells in the main simulation volume (where Purkinje cell somata and Bergmann glia are present, see panel E), and orange fibers are ingrowing parallel fibers. (B) Schematic representations of how each granule cell migrates using a Bergmann glia process as a guide, see text for explanation. (C) Histogram showing final lengths of the parallel fibers in the representative case, 300 μm is the maximum possible length for the simulation volume. (D) Bar plots showing success rates of soma radial migrations, of parallel fiber branching to form T-junctions, of parallel fiber extensions initiated in the main volume and of parallel fibers growing from the outside into the main volume and fully extending in the main volume. Mean success rates from 10 simulation samplings of the model with error bars showing standard deviations. Coefficient of variation values for each is 0.031, 0.007, 0.007, and 0.004 respectively. (E) Another side view (longitudinal side) of the granule cell model after all green granule cells finish migration. Black to gray stripes represent z-locations of granule cell births during 12 phases. The central part is the main simulation cube (panel A), left and right sides are origin sites of ingrowing parallel fibers. (F) Top view of the model with numbering of Purkinje cell somata. Brown cells were added to provide dendritic repulsion at the borders of the main simulation cube but were not analyzed. Blue structures are Bergmann somata and radial glia.

In order to provide a 3D environment for Purkinje dendrites to grow, a granule cell migration model was built first. The model simulates a small volume of space in which we estimate about 4,500 granule cells and 24 Purkinje cells [34] will be present at adult stage. As we only simulate P1 to P14, about 3,000 granule cells migrate in this volume and form parallel fibers (Fig 2A) and Purkinje cell dendrites do not grow. The model does not simulate tissue expansion. To approximate this, granule cells are born in 12 consecutive phases from bottom to top (Fig 2E), simulating the expansion of the molecular layer pushing the external granular layer upward. Parallel fibers are much longer than the width of this volume, therefore, we simulate an additional 10,000 parallel fibers growing in from neighboring volumes that do not simulate Purkinje cell or granule cell growth.

Fig 2B summarizes the sequences simulated during granule cell migration. First, a granule cell soma (small green dot) migrates horizontally to one of the closest Bergmann glia processes (light blue, more distant glia processes of same cell are dark blue) by extending a cylindrical leading process (gray). The granule cell will migrate following its leading process till it gets close enough to the target Bergmann glia process. There, it changes direction and starts a radial migration by extending the leading process downwards, staying close to the process. It also extends a short axon (pink) from its tail which will further bifurcate into parallel fibers. The soma keeps migrating down and parallel fibers further extend along the y-axis in both directions. A line of axonal fronts is laid down along the path of the radial migration of the soma.

In a representative model case, 80.59% of the granule cells succeeded in the radial migration, and 67.73% of parallel fibers extended through the complete volume of y-axis (Fig 2C). Because of crowding, only 29.31% of incoming parallel fibers completed extensions through the main volume. Total parallel fibers in the central volume was 3,487 towards front and 3,430 to back. Distributions of success rates from 10 simulations are shown in Fig 2D.

### Purkinje cell growth model

Purkinje cell growth in the model corresponds to development in a mouse from P4 to P14. It starts by growing five new dendrites on the upper sphere of each soma, of which four will be retracted in two phases, followed by a few days of growth of the winner tree (Fig 1B). The initiation of dendritic growth waits till early granule cell migrations are completed, and starts at initiation of phase 6 granule cell migrations (Fig 2E, cycle 65). To reduce overall run-times, 10–20 simulations of the early granule cell migration phases were saved and used as the common initial conditions for the different Purkinje cell control and retraction scenarios (except for NoGranCells).

We assumed that a major goal for Purkinje cell dendrite growth is to establish synaptic contacts with parallel fibers [16, 35, 36]. Therefore, dendrites grow toward nearby parallel fibers. Once they reach a parallel fiber, a synaptic contact is made and growth continues in a direction perpendicular to the parallel fiber [37, 38]. In addition, dendrites are repelled by other nearby dendrites and have a low probability of branching. The specific growth rules used are described in Materials and Methods. In NeuroDevSim, synapses do not have a physical structure but, because each front can only have one synapse, they can never be very close to each other. In most of the models, synapses are passive and used only to compare the effectiveness of dendritic growth on connectivity.

Two phases of retraction are implemented in all models [29]. In the first phase, three trees retract and the remaining two trees continue to grow. In the final phase a single winner tree is selected and the other one is retracted. Before exploring possible mechanisms that control the selection of winning dendrites, we performed three control scenarios to investigate the influence of dendritic selection processes and physical presence of granule cells on dendritic tree morphology.

### Purkinje cell retraction controls

**Fig 3:**
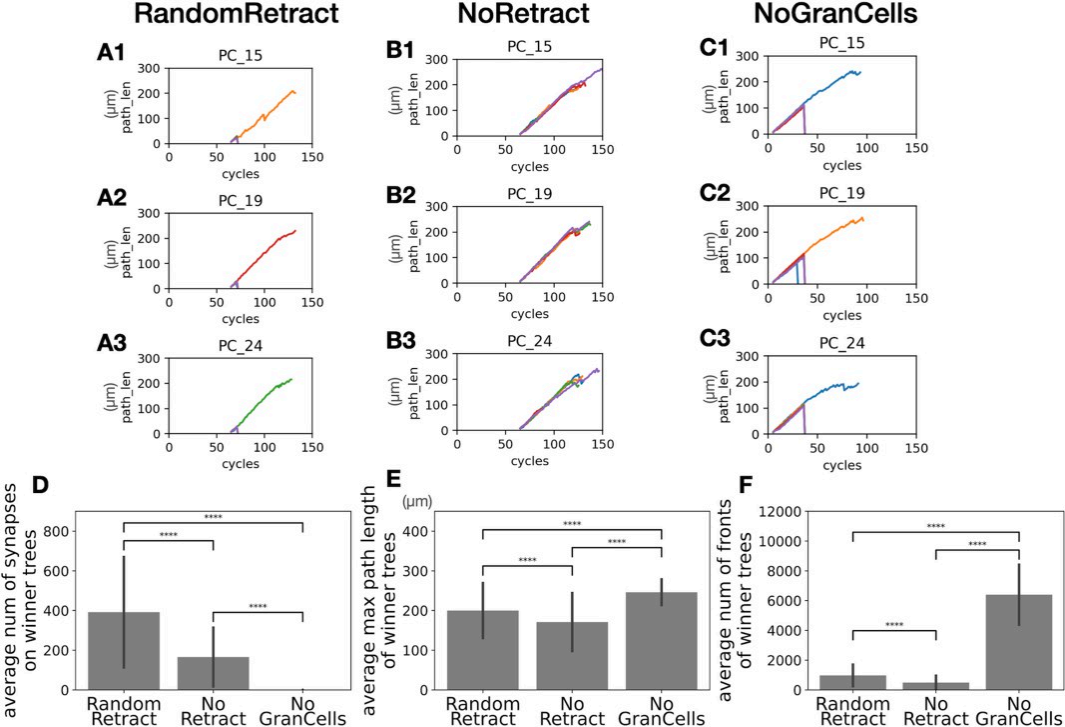
Characteristic of 3 control scenarios. (A1-3) The plots show changes in maximal path length of dendritic trees with a different line color for each tree. Three Purkinje cells were randomly selected from one of 10 RandomRetract simulations. (B1-3) Similar plots as A1-3 from one of 10 NoRetract simulations. (C1-3) Similar plots as A1-3 from one of 7 NoGranCells simulations. Here only C2 shows 2 phases of retraction. In C1 and C3, all dendritic trees at the first screening phase were larger than the retraction threshold and kept growing until the second screening phase. (D) Average number of synapses on each winner dendritic tree in the three scenarios with error bars representing standard deviations. (E) Average maximum length of each winner tree in the three scenarios with standard deviations. (F) Average number of branch points in each winner tree in the three scenarios with standard deviations. P values: **** indicates p < 0.00005, Welch’s *t*-test. Actual p values, t statistics, and degrees of freedom for comparing each data set are summarized in S1 Table.

**Fig 4:**
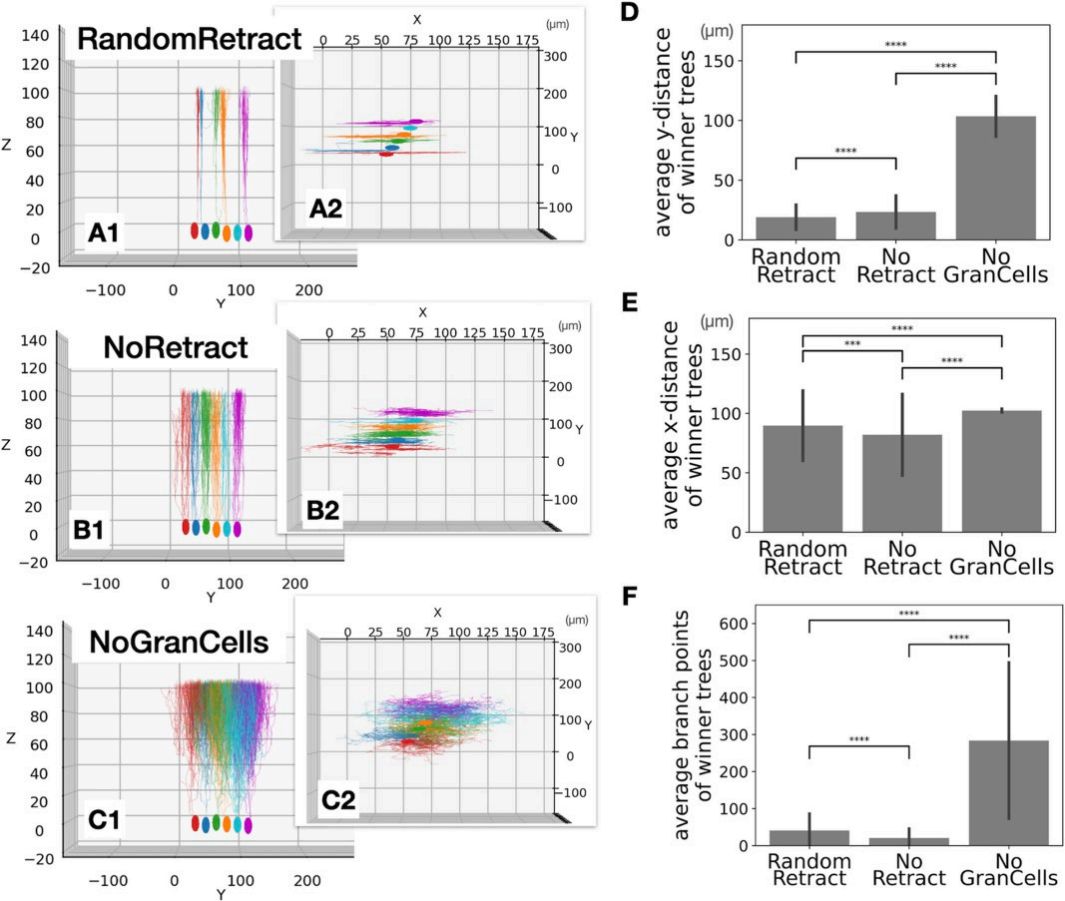
Arbor size of winner trees in 3 control scenarios. (A1) Side view of Purkinje cells at P12 (cycle 120) for RandomRetract scenario. Only Purkinje cells #21–26 are shown for convenience (see Fig 2F), while other structures in the simulation are hidden. (A2) A top view of A1. (B1) Similar plot for NoRetract scenario. (B2) A top view of B1. (C1) Similar plot for NoGranCells scenario. (C2) A top view of C1. (D) Average maximum y-distance for each scenario, error bars indicating standard deviations. (E) Average maximum x-distance for each scenario with standard deviations. (F) Average number of branch points for each scenario with standard deviations. P values: **** indicates p < 0.00005 and ** is p < 0.005, Welch’s *t*-test. D-E: 240, 240 and 168 cells. Actual p values, t statistics, and degrees of freedom for comparing each data set are listed in S2 and S3 Tables.

The control scenarios investigate how the growth rules affect Purkinje cell dendritic growth in the absence of specific retraction mechanisms. We simulated random selection of a winner, no retractions at all, or growth and retraction of dendrites in the absence of granule cells. Each of these scenarios is described in more detail below, and examples of dendritic growth and general comparisons are shown in Figs 3 and 4.

The RandomRetract scenario randomly chooses a single winner dendritic tree out of five candidates at 15 hours after start of growth (cycle 72) and retracts all others (visible at the beginning in each plot in Fig 3A1–3A3). The NoRetract scenario never initiates retractions and all the dendritic trees in each cell keep growing until they reach z = 100 μm (Fig 3B1–3B3). The NoGranCells scenario does not use the granule cell migration model. Instead, Purkinje cell somata are initiated in an empty volume of the same dimensions and dendritic growth is initiated at cycle 5. After 2.3 days of growth (cycle 30), cells that achieve a growth threshold retract small dendrites. 15 hours later (cycle 37), the tree with the largest number of fronts is selected as winner (Fig 3C1–3C3).

We next compare properties of the winner dendritic trees in the three control scenarios. The average number of synapses with parallel fibers in winner trees shows differences (Fig 3D). Most clearly, dendritic trees in NoGranCells did not have any synapses because there are no granule cells. Each winner dendritic tree in RandomRetract got a much larger number of synapses than in NoRetract, probably because growing all candidate trees in NoRetract led to more intense competition over limited numbers of synapse locations with parallel fibers. Moreover, in NoRetract, crowding by more dendritic branches in the simulation cube led to a decrease in the number of fully extended parallel fibers, which made the competition even more severe. Although experimental data to compare number of synapses on dendritic trees at the age of interest is not available, it seems the model provided enough opportunity for dendrites to form synapses with parallel fibers. Because retraction opens up space for growth of winner dendrites, they have a larger max path length and number of branch points (40.89 ± 45.2 versus 20.64 ± 25.2) in RandomRetract than in NoRetract (Fig 3E). This may also have contributed to the increase in number of synapses. Winner trees in NoGranCells had an artificially large increased number of total fronts (Fig 3F), due to how the Purkinje cell growth rule deals with an absence of nearby parallel fibers.

Examples of the morphology of the winner trees and a comparison of their dimensions are shown in Fig 4. Flat dendritic trees, measured by the maximum y-distance of winner trees, depend on the presence of granule cells (Fig 4A1–4C1 and 4D). Only RandomRetract and NoRetract show well separated planar morphologies, conversely NoGranCells dendritic trees intermingle each other. This indicates that repulsion by nearby dendrites [39], present in all three scenarios, was not sufficient to separate dendritic trees. Instead, growth along directions perpendicular to close by parallel fibers was more effective [37, 38] (see dendritic tree growth algorithm in Materials and Methods). Comparing the maximum y-distance of RandomRetract and NoRetract, RandomRetract has slightly thinner trees. Therefore, selection of a single winner tree seems to contribute to planar dendrites [20] and further growth of the winner tree did not result into expansion along the Y axis.

Although differences in maximum x-distance were less obvious, they were statistically significant (Fig 4A2–4C2 and 4E). The RandomRetract and NoRetract scenarios resulted in much greater variation between samples than NoGranCells. Physical hindrance from granule cells possibly contributed to enlarge the divergence in the tree morphologies.

In adult control mice, y-distance of dendritic trees was 13.9 ± 1.5 μm and x-distance was 82.9 ± 2.5 μm [40]. Y-distance from NoRetract and RandomRetract are slightly larger than this observation, though Purkinje cell dendritic trees attains single planar morphology only after P22 in mice [23] which is later than the simulated growth. For growth in x-axis direction, the dendritic tree reaches its full x-width roughly by P13 in mice [20]. The x- distance of the three scenarios was marginally wider than experimental observation [18]. Confirming the importance of lack of granule cells in NoGranCells, larger y-distances (28.7 ± 4.8 μm) were observed in reeler mice where the structure of cerebellar cortex is disrupted due to a failure of granule cell migration [40].

The simulation results from these scenarios suggest that excess trees growing from the same cell lead to a reduced dendritic tree morphology by competition for space and synapses (NoRetract scenario), implying an important role for the retraction stage. Also, the physical presence of granule cells contributes to the distinct planar structure of Purkinje cell dendritic trees.

### Possible mechanisms for Purkinje dendrite retraction

The mechanisms controlling the timing and selection of dendrites to retract are at present unknown, but neuronal and synaptic activity are required and retraction occurs in two stages [29]. In this study we simulated four simple retraction mechanisms and compare their outcomes, assuming that the number of parallel fiber synapses made by the winner trees is an appropriate measure of success [36]. In the FixedRetract scenario all Purkinje cells retract dendritic trees at the same times and winners are selected based on number of synapses on each tree. In the other scenarios, individual cells need to achieve a growth threshold before retractions are triggered, resulting in different retraction times, and winner trees are selected based on different criteria. In SizeRetract, the size of the entire dendritic tree, measured by its number of fronts, is used to control the timing of retractions and winners are selected based on the size of each tree. In SynapseRetract the number of synapses controls both timings of retraction and selection of winners. In the first three scenarios, the role of granule cells in promoting retraction is only structural by providing axons that can have synaptic contacts with Purkinje cells. In the final scenario, InputRetract, we assume that granule cell network activity also contributes. Specifically, granule cells have random firing rates, with larger firing rates for cells that completed migration to the granular layer and can receive synaptic input by mossy fibers (not simulated). The amplitude of synaptic input to Purkinje cells is granule cell firing rate dependent and integrated over time as a synaptic signal (see Materials and methods). The summed synaptic signals are used in InputRetract to control both retraction timings and selection of winner dendritic trees.

**Fig 5:**
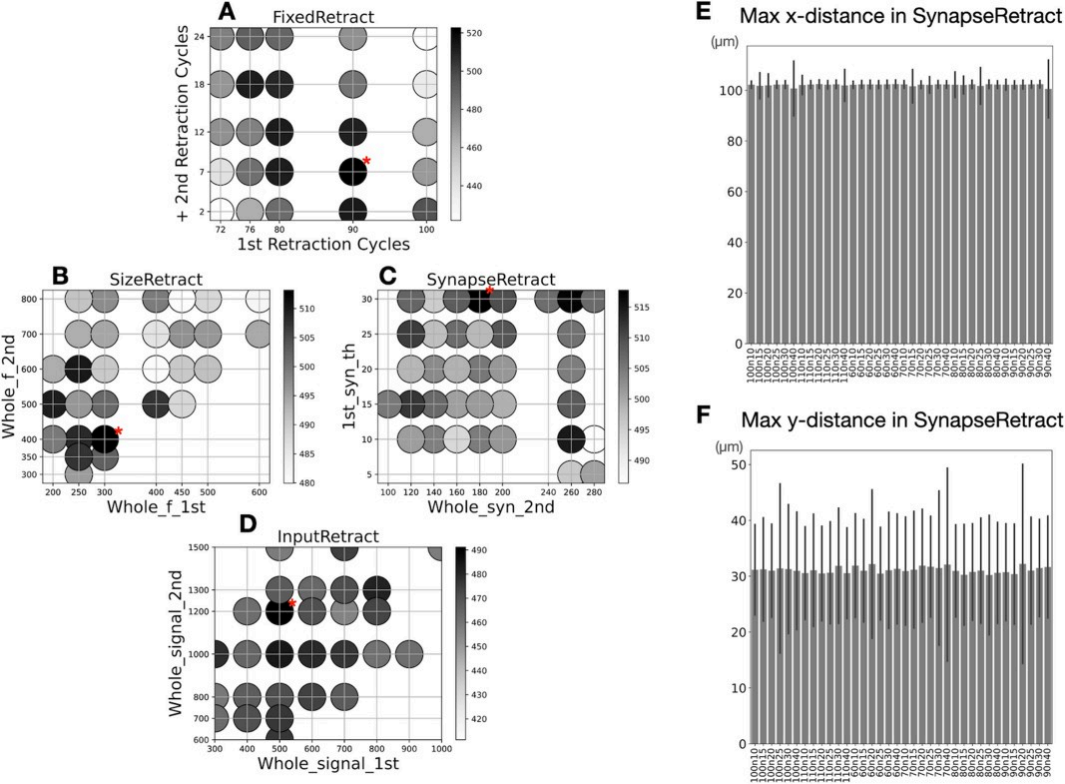
Dependence of number of synapses and arbor size of winner trees on parameter sets. The scatter maps show number of synapses by darkness for each combination of control parameters. The sizes of circles on the plot represent average maximum path length of winner trees. The parameter set with the most numbers of synapses is marked with a red asterisk, corresponding parameter values are listed in Table 1. (A) FixedRetract scenario: the control parameters are cycles at which each retraction phase occurs. X-axis shows the first retraction cycle and y-axis shows number of additional cycles to trigger second retraction. Each point represents 10 or 20 samples. (B) SizeRetract scenario: has 3 control parameters. X-axis shows number of fronts required to trigger first retraction and y-axis to trigger second retraction. In addition, during first retraction, a parameter (named ‘1st_f_th’ in Table 1) sets minimum number of fronts required to survive, 20 for this figure (range 20–160 evaluated). Each point represents 20 samples, surviving trees without synapses excluded. (C) SynapseRetract scenario: has 3 control parameters. X-axis shows total number of synapses required to trigger second retraction, and y-axis is minimum number of synapses required to survive first retraction. Total number of synapses required to trigger first retraction (named ‘1st_s_th’ in Table 1) is set at 90 (range 60–110 evaluated). Each point represents 20 samples. (D) InputRetract scenario: X-axis shows integrated synaptic signal required to trigger first retraction and y-axis does the same to trigger second retraction. Each point represents 10 to 20 samples. (E) Plotting average maximum x-distance of winner trees from all simulations of SynapseRetract with different parameter values as a representative example for all scenarios. Vertical lines indicate standard deviations. (F) The same plot as E, but for average maximum y-distance.

Each of the four retraction scenarios has two to three control parameters. In first instance, we performed parameter space evaluations to select the control parameter values that resulted in winner trees with the largest number of parallel fiber synapses (Fig 5 and Table 1).

**Table 1:** Summary of the data from the seven different scenarios. Number of synapses were counted per a cell if not indicated. Numbers of fronts, path length, number of branch points, number of terminals, max x-distance, max-y distance were counted per a winner tree.

	retraction parameter _1	retraction parameter_2	retraction parameter_3	number of synapses	number of fronts	path length	number of branch points	number of terminals	max x-distance	max y-distance	number of primary trees
RandomRetract	**random_retract_cycle = 72**	**N/A**	**N/A**	**391.6 ± 278.7**	**975.8 ± 726.5**	**199.8 ± 69.4**	**40.89 ± 45.2**	**41.23 ± 63.4**	**89.70 ± 29.5**	**19.01 ± 0.0569**	**Zero = 0;** **One = 240;** **Two = 0;** **More than 2 = 0**
NoRetract	**N/A**	**N/A**	**N/A**	**165.5±148.1** **(per a tree)** **813.6±285.9** **(per a cell)**	**496.11 ± 460.3**	**170.9 ± 73.1**	**20.64 ± 25.2**	**20.97 ± 33.2**	**82.03 ± 34.2**	**23.39 ± 0.0747**	**Zero = 0;** **One = 0;** **Two = 0;** **More than 2 = 240**
NoGranCells	**Whole_1st = 250**	**1st_f_th = 60**	**Whole_2nd = 400**	**N/A**	**6397.77 ± 2022.3**	**245.9 ± 32.6**	**283.77 ± 210.8**	**284.11 ± 328.4**	**102.35 ± 1.5**	**103.47 ± 0.0201**	**Zero = 0;** **One = 168;** **Two = 0;** **More than 2 = 0**
FixedRetract	**WholeCheck_cycle_1 = 90**	**WholeCheck_cycle_2 = 97**	**N/A**	**546.1 ± 201.7**	**1453.1 ± 588.2**	**226.3 ± 17.6**	**60.73 ± 47.3**	**61.07 ± 72.9**	**102.23 ± 3.1**	**31.31 ± 0.065**	**Zero = 0;** **One = 478;** **Two = 2;** **More than 2 = 0**
SizeRetract	**Whole_1st = 300**	**1st_f_th = 20**	**Whole_2nd = 400**	**525.0 ± 184.7**	**1395.41 ± 540.9**	**226.1 ± 16.0**	**59.22 ± 45.2**	**59.56 ± 70.1**	**102.32 ± 1.6**	**31.19 ± 0.05**	**Zero = 0;** **One = 479;** **Two = 1;** **More than 2 = 0**
SynapseRetract	**Whole_syn_1st = 90**	**1st_s_th = 30**	**Whole_syn_2nd = 180**	**530.4 ± 207.0**	**1402.5 ± 593.5**	**226.6 ± 16.2**	**59.55 ± 47.0**	**59.89 ± 72.3**	**102.46 ± 2.0**	**30.59 ± 0.0602**	**Zero = 1;** **One = 478;** **Two = 1;** **More than 2 = 0**
InputRetract	**Whole_signal_1st = 500**	**Whole_signal_2nd = 1,200**	**N/A**	**500.4 ± 203.5**	**1268.95 ± 572.1**	**224.42 ± 20.8**	**53.55 ± 43.3**	**53.88 ± 65.8**	**101.7 ± 6.0**	**28.59 ± 0.0838**	**Zero = 0;** **One = 480;** **Two = 0;** **More than 2 = 0**

There was a high variance in the number of synapses generated for different parameter combinations for all scenarios. While for scenarios FixedRetract and InputRetract clear trends were visible in the color maps, this was less the case for SizeRetract and SynapseRetract, resulting in noisier maps. Values for the best parameter sets (red stars in Fig 5) and average number of synapses on winners are listed in Table 1. Data from these best parameter sets were further analyzed in the following Figures.

Maximum x-distance and y-distance of winner trees were compared. Although especially y-distances showed great variability between cells, this was consistent for all parameter combinations (data for SynapseRetract shown in Fig 5E and 5F), and there were no differences with the measurements for other scenarios. Compared to experimental data [40], the same conclusions apply as for the measurements in the control scenarios (Fig 4E and 4F).

**Fig 6:**
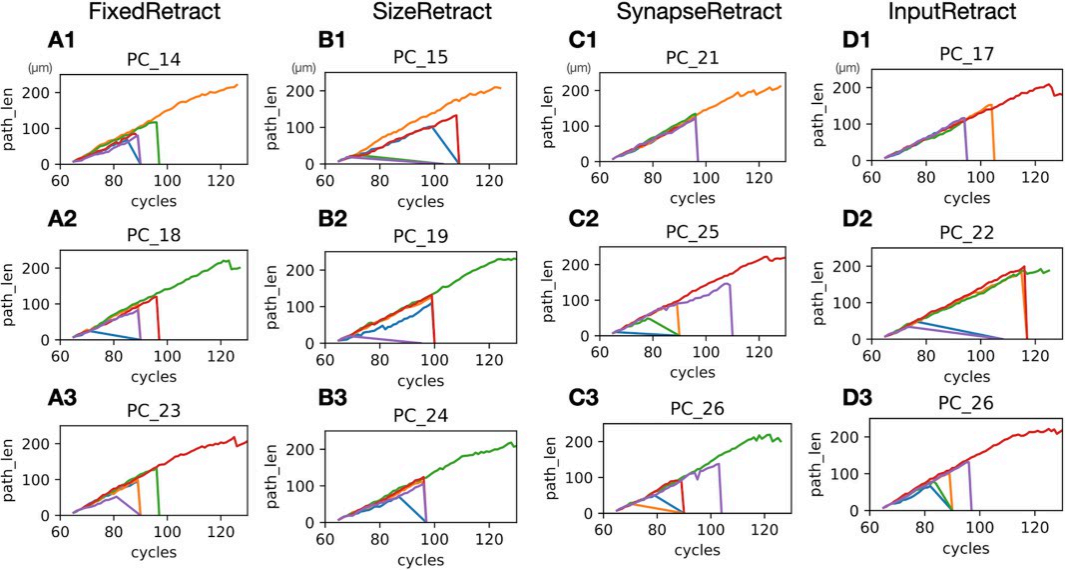
Change in path lengths with during simulation in retraction scenarios. The plots show changes in max path length of candidate trees at every simulation cycle with a different line color for each tree. Three Purkinje cells were randomly selected from one of 20 simulations. Max path lengths of a tree drops to 0 when they are retracted. Some trees stop growing before the retraction phase, resulting in more shallow lines, for example blue line in A2. (A1-3) FixedRetract scenario. Since this scenario induces retractions at two fixed cycles, all retractions occur at either cycle 90 or 97. (B1-3) SizeRetract scenario. Similar plots as A1-3. Some cells skipped the first retraction because all the dendritic trees had a larger number of fronts than the threshold (20 fronts) when a cell reached the first retraction threshold (300 fronts), resulting in a single retraction event. (C1-3) SynapseRetract scenario. (D1-3) InputRetract scenario. Plots were generated by data using parameters as in Table 1, except D1 and D2 are from a parameter set with Whole_signal_1st = 10,000 and Whole_signal_2nd = 13,000.

An important difference between the retraction scenarios is that FixedRetract forces two phased retractions at fixed cycles, while other scenarios can have retractions occur at different cycles for each cell (Fig 6).

**Fig 7:**
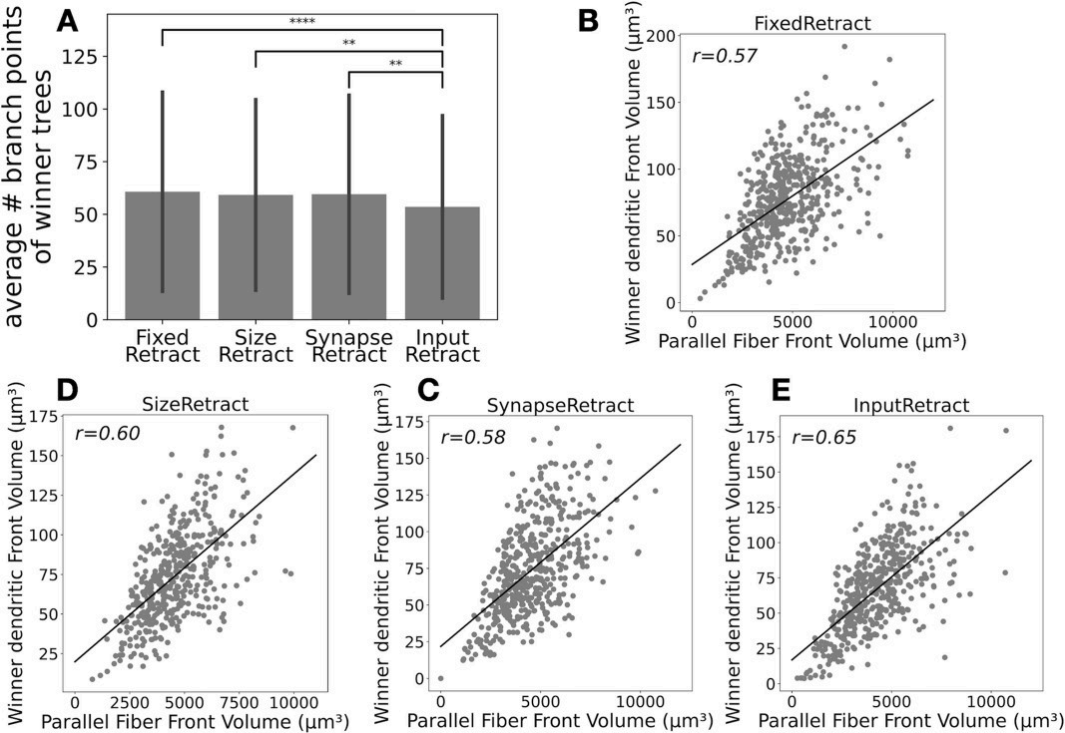
Size of winner trees in the retraction scenarios depends on local parallel fibers. (A) Average number of branch points on winner trees for the retraction scenarios with error bars indicating standard deviations. P values: * indicates p < 0.05 and ** is p < 0.005, Welch’s *t*-test. Actual p values, t statistics, and degrees of freedom for comparing each data set are listed in S3 Table. (B) FixedRetract: correlation between volume of all fronts of a winner tree and volume of all parallel fibers in the same neighborhood. (C) SizeRetract: similar correlation as in B. (D) SynapseRetract: similar correlation as in B. (E) InputRetract: similar correlation as in B.

Measurements that represent sizes of the winner trees, their number of fronts, branch points, and terminals, were strongly correlated with each other and therefore we show only the number of branch points (Fig 7A). InputRetract had significantly smaller trees than the other scenarios, which correlates with its smaller number of synapses (Table 1). Compared with control scenarios RandomRetract and NoRetract, winner trees from retraction scenarios have significantly more branch points (S3 Table), which suggests that proper selection of which trees to retract promotes growth of the winner dendritic trees. We found that the size of winner dendritic trees strongly correlates with the total volume of the parallel fibers in their direct neighborhood (Fig 7B–7E). This may be expected from the dendritic growth rule that promotes growth towards parallel fibers [38], but it also implies that the positive influence of parallel fibers is much larger than their negative crowding effect on growth.

**Fig 8:**
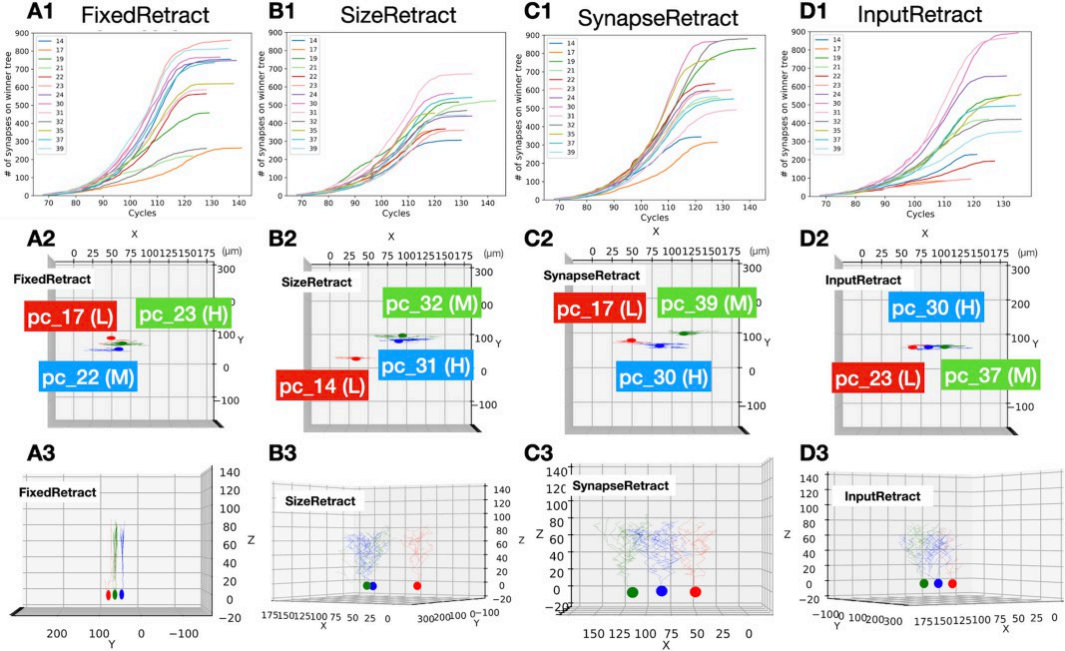
Changes in synapse numbers and morphology of individual winner trees from single samples in the retraction scenarios. (A1, B1, C1, and D1) Plots to show change in number of synapses at every simulation cycle in one of the simulations (seed 11). Colors refer to Purkinje cell numbers (see Fig 2F). (A2, B2, C2, and D2) Top view of selectively visualized Purkinje cells at P10 (cycle 100). Purkinje cells with highest (H), lowest (L), and medium (M) number of synapses from A1-D1 plots were selected for each. See Fig 2F for location of the cells. (A3, B3, C3, and D3) Plots to show morphology of winner trees in A2-D2 viewed from different directions.

Finally, we examined the variability of the number of synapses for the different retraction scenarios. In Fig 8 we show examples of how this variability develops over simulation time for different cells in each scenario. There were obvious differences between the scenarios in the range of number of synapses in the winner trees. The SizeRetract scenario clearly has a much lower variability (Fig 8B1), while that of InputRetract seems largest. Randomly selected Purkinje cells from these simulations are shown in A2-3, B2-3, C2-3, and D2-3. It is difficult to detect morphological differences between scenarios from the figures, but variation in morphology of Purkinje cell dendrites from the same simulations can be observed. Movies for the dendritic growths and retractions of selected cells in each scenario can be found in supplementary materials.

**Fig 9:**
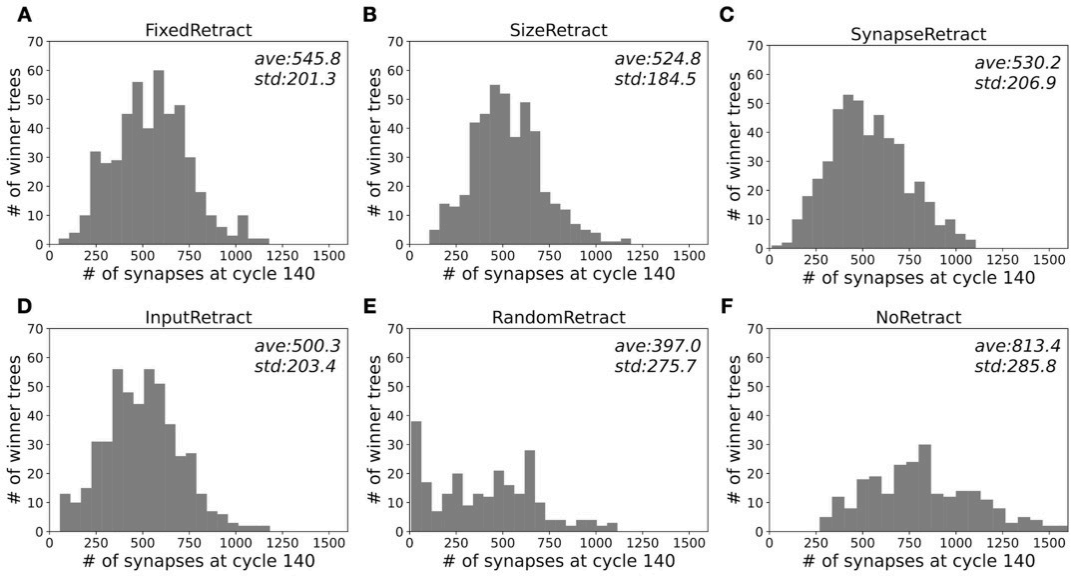
Variability of synapse numbers in all winner trees for the retraction scenarios. Histograms for numbers of synapses on winner trees from all 20 simulations in each retraction scenario, and 10 simulations in each control scenario. Average and standard deviation of each data set is shown in each plot. P values, t statistics, and degrees of freedom for comparing each data set are listed in S4 Table.

In Fig 9, the distributions of winner trees with a given number of synapses at the end of growth in all simulations are compared (See S4 Table for comparative statistics). All dendritic selection scenarios result in winner dendrites with significant higher number of synapses than RandomRetract, conversely NoRetract tends to have more synapses because it has 5 trees.

The variability in number of synapses of the InputRetract scenario (Fig 9D, Table 1) is larger than that of other retraction scenario (Fig 9A–9C) and this is mostly due to a larger proportion of winner trees with small numbers of synapses. This is caused by the input firing rate dependent selection rule of InputRetract, which favors winner trees that mainly synapse onto parallel fibers extending in the bottom of the molecular layer. The somata of the granule cells of these parallel fibers reach the granular layer first, strongly increasing their average firing rates.

## Discussion

### Summary and discussion of the main results

While it was challenging to achieve reasonable success rates for granule cell migration and parallel fiber growth in the very crowded environment of the molecular layer in the model (Fig 2), crowding was less of an issue for Purkinje cell dendritic growth. In fact, the presence of numerous parallel fibers was essential for planar growth of the Purkinje cell dendrite (Fig 4) and promoted the growth of larger dendritic trees (Fig 7). Conversely, dendrite-dendrite repulsion was not sufficient to generate flat dendrites. It should be noted, however, that these observations are emergent properties of the growth algorithms used, which were not varied systematically.

Comparisons of the control scenarios with the retraction ones allows to suggest possible roles of the dendritic selection stage. Winner trees based on selection were larger and had more synapses than those generated by RandomRetract (Figs 4, 7 and 9). They were also larger than those obtained in NoRetract (Figs 4 and 7), but no retraction did result in a larger number of synapses on the dendrite (Fig 9F).

The model then explored detailed mechanisms of dendritic selection comparing four retraction scenarios. In the absence of experimental data about the exact mechanisms controlling the selection of a winner dendrite, we assumed that an optimal developmental sequence generates the largest number of parallel fiber synapses onto a Purkinje cell dendritic tree. This is based on the uniquely high number of synaptic inputs on these neurons [36, 41] and on the effects of up- or down-regulation of parallel fiber synapses on dendritic morphology [23, 28]. We then devised scenarios that assume that the retraction process is primarily controlled by a genetic clock identical to all cells (FixedRetract), by size or synapse dependent Purkinje cell metabolic mechanisms resulting in variable retraction times (SizeRetract and SynapseRetract) or by network activity in the granule cell layer (InputRetract). Based on studies of the development of other brain regions [42, 43], the afferent network activity InputRetract scenario seemed the more plausible one. This would also match the late, postnatal development of cerebellar structures, which has been hypothesized to reflect the requirement of structured synaptic input to establish correct synaptic connectivity in cerebellum [44]. However, in our simulations, InputRetract was less successful than the other three scenarios, resulting in smaller winner trees with less synapses. This was due to a specific property of cerebellar development: the input granular layer is still forming while output Purkinje neurons grow their dendrites. A large fraction of early parallel fiber synapses will, therefore, be made onto axons of granule cells that have not yet reached their final position or had time to grow dendrites and establish mossy fiber contacts. In other words, around P6-P8 when dendritic tree retractions occur, the input layer is structurally very heterogenous, and this is probably reflected also in its firing rates. As a result, in the model, the InputRetract scenario selected winner trees that primarily made synapses in the lower region of the molecular layer. Conversely, the other retraction scenarios used number of synapses (FixedRetract and SynapseRetract) or dendritic size (SizeRetract) to select the winner tree and this resulted in less variable outcomes with more synapses (Fig 9). Therefore, we postulate that, in cerebellum, the dendritic tree selection cannot be based on afferent synaptic activity alone. Postsynaptic metabolic mechanisms that are sensitive to tree size and/or number of synapses are expected to play an important role, though we cannot include an additional effect of afferent input.

### Limitations of the early cerebellar development model

A major limitation for model development was the lack of experimental data. There are only a few reconstructions of normal Purkinje cell morphologies at P10-P14 available [45], insufficient for a systematic statistical comparison with the model morphologies [33, 46, 47]. Neither is data about the development and properties of parallel fiber synapses in normal mice at this critical age available.

The largest difference between the model and biological systems is that cerebellar expansion during development was not implemented, because volume expansion cannot be simulated in NeuroDevSim or any other neural development simulator [31, 48]. The simulator also does not support growth of front diameters. The cerebellum undergoes a massive expansion, especially along the anterior-posterior plane (x-axis of the simulation cube) [49] during the first two postnatal weeks in mice [50]. While the anterior-posterior plane in an embryonic days 17.5 mouse has a length of half the width of its molecular layer axis (z-axis of the cube), it becomes in postnatal mice 7.8 times longer than the molecular layer which also expands significantly [49]. The lack of volume expansion strongly increases crowding of cells, making it more difficult to simulate migration and growth of large cell populations. Growth may also stretch and remodel existing neural structures [31, 51], an effect not included in the simulations. It has been suggested that retraction of Purkinje cell branches may contribute to making the dendrite more planar, but this occurs at later ages (P18 to P25) than simulated here [23].

There is a difference in the process of extending parallel fibers during granule cell migration between the model and real neurons. In murine cerebellum, parallel fibers already extend during horizontal migration. However, the model extended parallel fiber axons after horizontal migration (Fig 2B). The purpose of horizontal migration in biological system is unclear, but it might be related to granule cell allocation upon expansion of the cortex [52], which is not implemented in the model. The model ignores the formation of synapses onto the descending part of the granule cell axon [53] and did not include development of inhibitory interneurons [54].

For Purkinje cell models, apoptosis and reorganizations of soma positions in Purkinje cell layer were not simulated. Also, the distinction between spiny dendrites and smooth dendrites is not made in the model. Climbing fibers and mossy fibers, two afferents to cerebellar cortex forming synapses with developing Purkinje cells [55, 56] are not included. Climbing fibers are involved in organizing the final shapes of Purkinje cell dendrites [57]. A reduced branching pattern in dendritic arborizations of Purkinje cells is observed in rats when climbing fibers were removed by thermocoagulation *in vivo* [58]. Similarly, transient contacts of mossy fibers with developing Purkinje cells are likely involved in assembly of zonal circuit maps of the cerebellum rather than dendritic selections [59, 60]. However, there is no strong evidence that they are involved in early dendritic development, and inclusions of these axons may be considered in future models. Finally, development was simulated only until P14, excluding later growth of the winning dendritic trees from analysis.

### Potential improvements and future directions of the models

In the model, all granule cells are homogeneous in that there is no zonal patterning by molecular phenotype or birth order [52]. Such inhomogeneity in granule cells can be represented by, for example, 2 different types of molecular diffusions in the model to facilitate the zoning. Also, we can set different types of granule cell objects and assign different preferences in making synapses with distal or proximal regions of Purkinje cells’ dendritic trees as observed [53, 61]. It is technically also possible to include granule cell proliferation, but in the absence of tissue expansion in the model this did not seem useful.

For the dendritic structure of Purkinje cells, an additional growth algorithm to facilitate elaboration of the proximal region of dendritic trees should be introduced to match observed morphologies. Also, while dendrite to dendrite repulsion was included in the growth algorithm [39], it was not studied systematically nor did we distinguish auto-repulsion from repulsion by other cells. Neither was the possible attraction of dendrites by Bergmann glia implemented [62], though one cannot exclude that the anatomical proximity reported was due to nearby descending granule cell axons instead of glia itself. Recent work on local versus global effects on dendritic morphology [63] may also provide a useful framework to analyze the typical branching patterns of Purkinje cell dendrites. In that regard, our Purkinje cell morphologies are quite similar to those of Luczak [64], which suggests that the parallel fibers in our model act as ‘neurotrophic particles’.

The present model is the first attempt to reconstruct early development of the main neurons in the cerebellar cortex with detailed interactions between several types of cells. The real cerebellum has evolved to be very complex, and experimental approaches to reveal how it develops are obviously important but limited by technical constraints. Modeling helps to re-organize and synthetize the experimental data and facilitates to understand how development works at different time scales.

## Materials and methods

The NeuroDevSim software version 1.0 [30] was used to construct and simulate the models (https://github.com/CNS-OIST/NeuroDevSim). Simulations were executed on the high-performance computing cluster Deigo at OIST, using AMD Epyc 7702 CPUs with 128 cores and 512GB memory. 64 cores were used for each simulation, and it took about 3 hours to finish a single simulation. Python scripts for each model can be found in the Model Database (https://modeldb.science/267591). Python notebooks and scripts and NeuroDevSim database files are available at https://zenodo.org/record/7771504.

The model embodies a Purkinje cell layer and a molecular layer in a central volume of (x1, y1, z1), (x2, y2, z2) = (-20, -20, -20), (180, 160, 140) μm^3^, representing a part of a murine cerebellar cortex (lobule VI) during postnatal days 1 to 14. The x-axis of the cube represents the sagittal plane, y-axis represents the transversal plane (long axis of folium), and z-axis is the depth of the cerebellar cortex (Fig 2A and 2E). The volume of the cube is fixed, therefore cerebellar tissue expansion is not implemented in the model.

The Purkinje cell layer has somas of Purkinje cells and Bergmann glia. The molecular layer has simplified Bergmann processes for guiding granule cells which gradually fill up the layer with their descending axons and parallel fibers (Fig 1A) and growing Purkinje cell dendrites (Fig 1B). Additionally, we simulated the in-growth of parallel fibers from neighboring regions, resulting in a total simulation volume of (-20, -160, -20), (180, 300, 140) μm^3^.

All growth follows a standard NeuroDevSim procedure: the end position of a new front is calculated and this is created as a new child, provided it does not collide with existing structures (Algorithm 1 in S1 Fig). In case of collision different solutions are used, dependent on which structure grows.

### Bergmann glia

On first cycle each Bergmann soma generates seven 4 μm long root processes that are spread along the x-axis. These then grow upward in 4 μm steps till they reach z = 131 μm (width of molecular layer at P11 [65] at cycle 35). Because Bergmann glia are first to grow, collisions are not an issue.

### Granule cell migration model

About 3,000 granule cells are gradually initiated in 12 consecutive phases in the central volume (Fig 2E) along with incoming parallel fibers of 10,000 granule cells located outside this central volume (orange structures in Fig 2E). Each phase initiates around 250 granule cells in the central volume and about 850 in-growing parallel fibers at the neighboring volumes. Upon initiation, granule cells migrate horizontally to a nearby Bergmann glia process (Fig 2B) and start downward radial migrations after they get close enough to the process (Fig 2B) (Algorithms 2 in S1 Fig and 3 in S2 Fig). During the radial migration, each granule cell soma extends an axon which further bifurcates as parallel fibers (Fig 2B). The cells keep migrating until they reach the internal granule layer (z = -15μm), while parallel fibers extend up to the ends of y-axis (Fig 2B). Granule cells start with a low random firing rate (0–0.2 / cycle) that strongly increases when they arrive in the internal granule layer (0–10 / cycle). The incoming parallel fiber structures simulate only parallel fibers themselves as a non-migrating soma with fibers extending along the positive or negative y-axis.

Granule cells in the model often experience collisions with surrounding structures during their migrations and extensions of axonal fibers. Built-in methods in NeuroDevSim are used to manage collisions; providing a bypass (*solve_collision*()) for migration, and finding an alternative space around a colliding structure (*alternate_location*()) for the fiber extensions. Also, the granule cell soma radius in the model is smaller than the actual value since physical shoving between cells cannot be simulated in the model, the smaller diameter representing the ‘squeezed’ soma structure of actual cells.

### Purkinje cell model

#### Growth algorithm

The soma activates on cycle 65 (Algorithm 4 in S3 Fig) and grows 5 dendritic roots (Algorithm 5 in S3 Fig).

The basic growth algorithm (Algorithm 6 in S4 Fig) simulates growth of a tip of dendrite towards a nearby synapse free parallel fiber segment. Once it gets close to the target, it makes a synapse and continues to grow perpendicular to the parallel fiber as observed in biological systems [37, 38]. If no free parallel fiber segment is nearby, the dendrites continue growth along the direction of their current heading with a small upward force assuming phenomenological involvement of interneurons in the molecular layers [66]. When dendrites experience collisions, the code use the built-in *solve_collision*() method to find a detour to reach a destined position. Repulsion force by the closest dendritic tree of other cells or of the same cell also affect the growth direction, with strength of repulsion depending on distance along the y-axis to the closest dendrite segment (Algorithm 6 in S4 Fig). During the elongation process, random branching events occur with a small probability (Algorithm 7 in S5 Fig). A dendrite tip also initiates a branch towards its target parallel fiber if the direction to it strongly deviates from its current heading direction (Algorithm 8 in S5 Fig).

## Control scenarios

Three control scenarios were simulated:

- **RandomRetract**: Purkinje cells skip the dendritic selection stage by randomly selecting a single primary tree out of 5 candidate trees for each Purkinje cell 7 cycles after initiating growth.
- **NoRetract**: never triggers retractions of the candidate trees resulting in a physically more crowded environment and increased competition for synaptic locations.
- **NoGranCells**: Purkinje cells grow in an empty cube without granule cell migrations. The retraction mechanism for this scenario used that of the **SizeRetract** scenario so that they can initiate retractions without granule cells. At cycle 30 any cell with more than 250 fronts will retract trees that have less than 60 fronts. At cycle 37 cells having at least 400 fronts will select their largest tree and retract all others.

## Retraction Scenarios

To select the surviving dendrite, four different retraction scenarios are implemented in the model:

- **FixedRetract** triggers dendritic retractions at fixed cycles. It uses two fixed simulation cycles to trigger retractions. At first cycle, three out of 5 trees are retracted and later the two remaining compete to become winner(s). Winner trees are determined by relative numbers of synapses for each dendritic tree in a cell. Parameters: the two cycles at which to retract, same for all cells.
- **SizeRetract** uses an internal parameter of growth, the size of each branch measured as its number of fronts. Granule cells merely acts as physical obstacles to dendritic growth. Cycles at which to retract are determined by cell thresholds for the summed sizes of all trees and vary between cells. At first retraction any tree smaller than a tree threshold is retracted, as a consequence a variable number of trees remains. Parameters: two size cell thresholds to start retraction and tree threshold.
- **SynapseRetract** uses the presence of synaptic connectivity. Cycles at which to retract are determined by cell thresholds for the number of synapses with parallel fibers of all trees and vary between cells. At first retraction any tree with less parallel fiber synapses than a tree threshold is retracted, as a consequence a variable number of trees remains. Parameters: two synapse cell thresholds to start retraction and tree threshold.
- **InputRetact** uses synaptic input determined by granule network activity levels. Synaptic input is integrated as an input signal proportional to afferent firing rate (equation in Algorithm 4 in S3 Fig) and relative total signal per tree is used to determine winner trees. Cycles at which to retract are determined by cell thresholds for total signal summed over all trees and vary between cells. Parameters: two signal cell thresholds.

## Data analysis

24 Purkinje cells enclosed by outer Purkinje cells were sampled for analysis (Fig 2F). For all scenarios, morphology of resulted dendritic trees was analyzed and compared. Pairwise statistics were made using the Welch’s t-test.

The following properties were computed:

- number of winner trees
- size of winner trees: computed as number of fronts because dendritic fronts tend to have uniform length of ~ 5 μm
- number of synapses
- maximum x- and maximum y-distance: computed for each tree as difference between minimal and maximal x or y coordinate value for the end point of all fronts
- number of branch and terminal points of winner trees.

The correlation analysis in Fig 7B–7E used the following algorithm:

- compute bounding box around Purkinje cell as minimal and maximal x or y coordinate value for the origin and end points of all dendritic fronts, with z in 17–100 μm
- sum volume of all cylindrical Purkinje dendrite fronts
- detect all parallel fiber fronts that have origin and/or end point in bounding box, compute their volume for the part that is contained in the box and sum
- correlate the two summed volumes for all cells.

## Supporting information

The Tables summarize comparisons of statistics from different retraction scenarios. P values (indicated as “p = ”) and T statistics (“t = ”) were calculated based on Welch’s t-test: **** indicates p < 0.00005, *** is p < 0.0005, ** is p < 0.005, and * is p < 0.05. Degree of freedom are shown as “df = ”.

S1 Table Statistics for comparing data in Fig 3D, 3E and 3F. (TIFF)

S2 Table Statistics for comparing data in Fig 4D and 4E. (TIFF)

S3 Table Statistics for comparing data in Fig 9 (Number of synapses on winner trees). (TIFF)

S4 Table Statistics for comparing data in Figs 4F and 7A (average number of branchpoints on winner trees). (TIFF)

All movies show only a few Purkinje cells: cells 21 in red, 24 in blue, 29 in green, and 33 in yellow (see Purkinje cell location map on Fig 4D). Granule cells, parallel fibers and Bergmann glia are not drawn. For each Purkinje cell, the dendritic trees in color are final primary trees, while ones that will be retracted are drawn in black. The movies start from simulation cycle 61 and ends at cycle 140.

S1 Movie Dendritic growths and retractions movie for control scenario RandomRetract (grow one tree). Seed 2 of scenario RandomRetract simulation was used to generate the movie.
(MP4)

S2 Movie Dendritic growths and retractions movie for control scenario NoRetract (grow all trees). Seed 1 of NoRetract scenario simulation was used to generate the movie.
(MP4)

S3 Movie Dendritic growths and retractions movie for control scenario NoGranCells (no granule cells). Seed 1 of NoGranCells scenario simulation was used to generate the movie, this movie starts from simulation cycle 0 and ends at cycle 140.
(MP4)

S4 Movie Dendritic growths and retractions movie for retraction scenario FixedRetract (retract by fixed timings). Seed 1 of retraction FixedRetract scenario simulation was used to generate the movie.
(MP4)

S5 Movie Dendritic growths and retractions movie for retraction scenario SizeRetract (retract by size maturation). Seed 1 of retraction SizeRetract scenario simulation was used to generate the movie.
(MP4)

S6 Movie Dendritic growths and retractions movie for retraction scenario SynapseRetract (retract by synaptic maturation). Seed 11 of retraction SynapseRetract scenario simulation was used to generate the movie.
(MP4)

S7 Movie Dendritic growths and retractions movie for retraction InputRetract (retract by maturation of network activity). Seed 11 of retraction InputRetract scenario simulation was used to generate the movie.
(MP4)

S1 Fig Algorithm 1: Single front growth and Algorithm 2: Manage granule cell fronts. (TIFF)

S2 Fig Algorithm 3: gc_soma. (TIFF)

S3 Fig Algorithm 4: Manage Purkinje cell fronts and Algorithm 5: Dendritic root initialization. (TIFF)

S4 Fig Algorithm 6: Dendritic elongation. (TIFF)

S5 Fig Algorithm 7: Branching and Algorithm 8: Directed branching. (TIFF)

## References

[1] Limperopoulos C, Bassan H, Gauvreau K, Robertson RL Jr, Sullivan NR, Benson CB, et al. Does cerebellar injury in premature infants contribute to the high prevalence of long-term cognitive, learning, and behavioral disability in survivors? Pediatrics. 2007;120(3):584–93. doi: 10.1542/peds.2007-1041 17766532

[2] Tavano A, Grasso R, Gagliardi C, Triulzi F, Bresolin N, Fabbro F, et al. Disorders of cognitive and affective development in cerebellar malformations. Brain. 2007;130(10):2646–60. doi: 10.1093/brain/awm201 17872929

[3] Manto M, Gruol DL, Schmahmann JD, Koibuchi N, Rossi F. Handbook of the cerebellum and cerebellar disorders: Springer; 2013.

[4] Butts T, Green MJ, Wingate RJ. Development of the cerebellum: simple steps to make a ‘little brain’. Development. 2014;141(21):4031–41. doi: 10.1242/dev.106559 25336734

[5] Bastian AJ, Martin T, Keating J, Thach W. Cerebellar ataxia: abnormal control of interaction torques across multiple joints. Journal of neurophysiology. 1996;76(1):492–509. doi: 10.1152/jn.1996.76.1.492 8836239

[6] Zanjani H, Vogel M, Delhaye-Bouchaud N, Martinou J, Mariani J. Increased inferior olivary neuron and cerebellar granule cell numbers in transgenic mice overexpressing the human Bcl-2 gene. Journal of neurobiology. 1997;32(5):502–16. doi: 10.1002/(sici)1097-4695(199705)32:5&lt;502::aid-neu5&gt;3.0.co;2-9 9110261

[7] Steen A-B. Quantitative Morphological Analyses of the striatum and cerebellum of Tenascin-R deficient mice: Staats-und Universitätsbibliothek Hamburg Carl von Ossietzky; 2006.

[8] Keller D, Ero C, Markram H. Cell Densities in the Mouse Brain: A Systematic Review. Front Neuroanat. 2018;12:83. doi: 10.3389/fnana.2018.00083 30405363 PMC6205984

[9] Xu H, Yang Y, Tang X, Zhao M, Liang F, Xu P, et al. Bergmann glia function in granule cell migration during cerebellum development. Mol Neurobiol. 2013;47(2):833–44. doi: 10.1007/s12035-013-8405-y 23329344

[10] Rakic P. Neuron-glia relationship during granule cell migration in developing cerebellar cortex. A Golgi and electonmicroscopic study in Macacus rhesus. Journal of Comparative Neurology. 1971;141(3):283–312. 4101340 10.1002/cne.901410303

[11] Hatten ME. Central nervous system neuronal migration. Annual review of neuroscience. 1999;22(1):511–39. doi: 10.1146/annurev.neuro.22.1.511 10202547

[12] Husmann K, Faissner A, Schachner M. Tenascin promotes cerebellar granule cell migration and neurite outgrowth by different domains in the fibronectin type III repeats. The Journal of cell biology. 1992;116(6):1475–86. doi: 10.1083/jcb.116.6.1475 1371773 PMC2289382

[13] Hatten ME. Riding the glial monorail: a common mechanism for glialguided neuronal migration in different regions of the developing mammalian brain. Trends in neurosciences. 1990;13(5):179–84. doi: 10.1016/0166-2236(90)90044-b 1693236

[14] Cuntz H, Mathy A, Häusser M. A scaling law derived from optimal dendritic wiring. Proceedings of the National Academy of Sciences. 2012;109(27):11014–8. doi: 10.1073/pnas.1200430109 22715290 PMC3390826

[15] Cuntz H, Borst A, Segev I. Optimization principles of dendritic structure. Theoretical Biology and Medical Modelling. 2007;4:1–8. 17559645 10.1186/1742-4682-4-21 PMC1924501

[16] Cuntz H, Forstner F, Borst A, Häusser M. One rule to grow them all: a general theory of neuronal branching and its practical application. PLoS computational biology. 2010;6(8):e1000877. doi: 10.1371/journal.pcbi.1000877 20700495 PMC2916857

[17] Wen Q, Stepanyants A, Elston GN, Grosberg AY, Chklovskii DB. Maximization of the connectivity repertoire as a statistical principle governing the shapes of dendritic arbors. Proceedings of the National Academy of Sciences. 2009;106(30):12536–41. doi: 10.1073/pnas.0901530106 19622738 PMC2713752

[18] Cajal RS. Histologie du système nerveux de l’Homme et des Vertébrés. Grand sympathique. Paris Maloine. 1911;2:891–942.

[19] Armengol J-A, Sotelo C. Early dendritic development of Purkinje cells in the rat cerebellum. A light and electron microscopic study using axonal tracing in ‘in vitro’slices. Developmental brain research. 1991;64(1–2):95–114. doi: 10.1016/0165-3806(91)90213-3 1786652

[20] Sotelo C, Dusart I. Intrinsic versus extrinsic determinants during the development of Purkinje cell dendrites. Neuroscience. 2009;162(3):589–600. doi: 10.1016/j.neuroscience.2008.12.035 19166910

[21] Fujishima K, Horie R, Mochizuki A, Kengaku M. Principles of branch dynamics governing shape characteristics of cerebellar Purkinje cell dendrites. Development. 2012;139(18):3442–55. doi: 10.1242/dev.081315 22912417 PMC3491647

[22] McKay BE, Turner RW. Physiological and morphological development of the rat cerebellar Purkinje cell. J Physiol. 2005;567(Pt 3):829–50. doi: 10.1113/jphysiol.2005.089383 16002452 PMC1474219

[23] Kaneko M, Yamaguchi K, Eiraku M, Sato M, Takata N, Kiyohara Y, et al. Remodeling of monoplanar Purkinje cell dendrites during cerebellar circuit formation. PLoS One. 2011;6(5):e20108. doi: 10.1371/journal.pone.0020108 21655286 PMC3105010

[24] Tanaka M, Yanagawa Y, Obata K, Marunouchi T. Dendritic morphogenesis of cerebellar Purkinje cells through extension and retraction revealed by long-term tracking of living cells in vitro. Neuroscience. 2006;141(2):663–74. doi: 10.1016/j.neuroscience.2006.04.044 16730917

[25] Das GD, Lammert GL, McAllister JP. Contact guidance and migratory cells in the developing cerebellum. Brain Research. 1974;69(1):13–29. doi: 10.1016/0006-8993(74)90366-7 4817907

[26] Ono K, Shokunbi T, Nagata I, Tokunaga A, Yasui Y, Nakatsuji N. Filopodia and growth cones in the vertically migrating granule cells of the postnatal mouse cerebellum. Experimental brain research. 1997;117(1):17–29. doi: 10.1007/pl00005787 9386001

[27] Otero JJ, Kalaszczynska I, Michowski W, Wong M, Gygli PE, Gokozan HN, et al. Cerebellar cortical lamination and foliation require cyclin A2. Dev Biol. 2014;385(2):328–39. doi: 10.1016/j.ydbio.2013.10.019 24184637 PMC3909955

[28] Takeo YH, Shuster SA, Jiang L, Hu MC, Luginbuhl DJ, Rülicke T, et al. GluD2-and Cbln1-mediated competitive interactions shape the dendritic arbors of cerebellar Purkinje cells. Neuron. 2021;109(4):629–44.e8. doi: 10.1016/j.neuron.2020.11.028 33352118 PMC8833808

[29] Takeo YH, Miura E, Yuzaki M. In vivo dendritic development of cerebellar Purkinje cells [Society for Neuroscience abstract]. San Diago, CA2017 [https://www.abstractsonline.com/pp8/#!/4376/presentation/7158. 10.1523/JNEUROSCI.0075-15.2015 PMC6605396 26354918

[30] De Schutter E. Efficient simulation of neural development using shared memory parallelization. Frontiers in Neuroinformatics. 2023. 10.3389/fninf.2023.1212384 PMC10400717 37547492

[31] Koene RA, Tijms B, Van Hees P, Postma F, De Ridder A, Ramakers GJ, et al. NETMORPH: a framework for the stochastic generation of large scale neuronal networks with realistic neuron morphologies. Neuroinformatics. 2009;7:195–210. doi: 10.1007/s12021-009-9052-3 19672726

[32] Ascoli GA, Krichmar JL, Nasuto SJ, Senft SL. Generation, description and storage of dendritic morphology data. Philosophical Transactions of the Royal Society of London Series B: Biological Sciences. 2001;356(1412):1131–45. doi: 10.1098/rstb.2001.0905 11545695 PMC1088507

[33] Kanari L, Dictus H, Chalimourda A, Arnaudon A, Van Geit W, Coste B, et al. Computational synthesis of cortical dendritic morphologies. Cell Reports. 2022;39(1):110586. doi: 10.1016/j.celrep.2022.110586 35385736

[34] Vogel MW, Sunter K, Herrup K. Numerical matching between granule and Purkinje cells in lurcher chimeric mice: a hypothesis for the trophic rescue of granule cells from target-related cell death. Journal of Neuroscience. 1989;9(10):3454–62. doi: 10.1523/JNEUROSCI.09-10-03454.1989 2795133 PMC6569898

[35] Chklovskii DB. Synaptic connectivity and neuronal morphology: two sides of the same coin. Neuron. 2004;43(5):609–17. doi: 10.1016/j.neuron.2004.08.012 15339643

[36] Harvey R, Napper R. Quantitatives studies on the mammalian cerebellum. Progress in neurobiology. 1991;36(6):437–63. 1947173 10.1016/0301-0082(91)90012-p

[37] Nagata I, Ono K, Kawana A, Kimura-Kuroda J. Aligned neurite bundles of granule cells regulate orientation of Purkinje cell dendrites by perpendicular contact guidance in two-dimensional and three-dimensional mouse cerebellar cultures. Journal of Comparative Neurology. 2006;499(2):274–89. doi: 10.1002/cne.21102 16977618

[38] Fujishima K, Kurisu J, Yamada M, Kengaku M. βIII spectrin controls the planarity of Purkinje cell dendrites by modulating perpendicular axon-dendrite interactions. Development. 2020;147(24):dev194530. 33234719 10.1242/dev.194530

[39] Fujishima K, Kawabata Galbraith K, Kengaku M. Dendritic self-avoidance and morphological development of cerebellar purkinje cells. The Cerebellum. 2018;17:701–8. doi: 10.1007/s12311-018-0984-8 30270408

[40] Kim J, Kwon N, Chang S, Kim KT, Lee D, Kim S, et al. Altered branching patterns of Purkinje cells in mouse model for cortical development disorder. Sci Rep. 2011;1:122. doi: 10.1038/srep00122 22355639 PMC3216603

[41] Kurihara H, Hashimoto K, Kano M, Takayama C, Sakimura K, Mishina M, et al. Impaired parallel fiber→ Purkinje cell synapse stabilization during cerebellar development of mutant mice lacking the glutamate receptor δ2 subunit. Journal of Neuroscience. 1997;17(24):9613–23. 9391016 10.1523/JNEUROSCI.17-24-09613.1997 PMC6573399

[42] Espinosa JS, Stryker MP. Development and plasticity of the primary visual cortex. Neuron. 2012;75(2):230–49. doi: 10.1016/j.neuron.2012.06.009 22841309 PMC3612584

[43] Yang J-W, Kilb W, Kirischuk S, Unichenko P, Stüttgen MC, Luhmann HJ. Development of the whisker-to-barrel cortex system. Current opinion in neurobiology. 2018;53:29–34. doi: 10.1016/j.conb.2018.04.023 29738998

[44] Verduzco-Flores SO, De Schutter E. Self-configuring feedback loops for sensorimotor control. Elife. 2022;11:e77216. doi: 10.7554/eLife.77216 36373657 PMC9699696

[45] Jayabal S, Ljungberg L, Watt AJ. Transient cerebellar alterations during development prior to obvious motor phenotype in a mouse model of spinocerebellar ataxia type 6. The Journal of physiology. 2017;595(3):949–66. doi: 10.1113/JP273184 27531396 PMC5285638

[46] Kanari L, Dłotko P, Scolamiero M, Levi R, Shillcock J, Hess K, et al. A topological representation of branching neuronal morphologies. Neuroinformatics. 2018;16:3–13. doi: 10.1007/s12021-017-9341-1 28975511 PMC5797226

[47] Scorcioni R, Polavaram S, Ascoli GA. L-Measure: a web-accessible tool for the analysis, comparison and search of digital reconstructions of neuronal morphologies. Nature Protocols. 2008;3(5):866–76. doi: 10.1038/nprot.2008.51 18451794 PMC4340709

[48] Zubler F, Douglas R. A framework for modeling the growth and development of neurons and networks. Front Comput Neurosci. 2009;3:25. doi: 10.3389/neuro.10.025.2009 19949465 PMC2784082

[49] Legue E, Riedel E, Joyner AL. Clonal analysis reveals granule cell behaviors and compartmentalization that determine the folded morphology of the cerebellum. Development. 2015;142(9):1661–71. doi: 10.1242/dev.120287 25834018 PMC4419279

[50] Altman J, Bayer S. Development of the cerebellar system: In relation to its evolution, structure, and functions CRC Press. Boca Raton, FL [Google Scholar]. 1997.

[51] Baltruschat L, Tavosanis G, Cuntz H. A developmental stretch-and-fill process that optimises dendritic wiring. BioRxiv. 2020:2020.07.07.191064.

[52] Consalez GG, Goldowitz D, Casoni F, Hawkes R. Origins, development, and compartmentation of the granule cells of the cerebellum. Frontiers in Neural Circuits. 2021;14:611841. doi: 10.3389/fncir.2020.611841 33519389 PMC7843939

[53] Gundappa-Sulur G, De Schutter E, Bower JM. Ascending granule cell axon: an important component of cerebellar cortical circuitry. Journal of Comparative Neurology. 1999;408(4):580–96. doi: 10.1002/(sici)1096-9861(19990614)408:4&lt;580::aid-cne11&gt;3.0.co;2-o 10340507

[54] Dorgans K, Demais V, Bailly Y, Poulain B, Isope P, Doussau F. Short-term plasticity at cerebellar granule cell to molecular layer interneuron synapses expands information processing. Elife. 2019;8:e41586. doi: 10.7554/eLife.41586 31081751 PMC6533085

[55] Altman J. Postnatal development of the cerebellar cortex in the rat. II. Phases in the maturation of Purkinje cells and of the molecular layer. Journal of Comparative Neurology. 1972;145(4):399–463. doi: 10.1002/cne.901450402 5044254

[56] Takeda T, Maekawa K. Transient direct connection of vestibular mossy fibers to the vestibulocerebellar Purkinje cells in early postnatal development of kittens. Neuroscience. 1989;32(1):99–111. doi: 10.1016/0306-4522(89)90110-3 2586754

[57] Berry M, Bradley P. The growth of the dendritic trees of Purkinje cells in the cerebellum of the rat. Brain research. 1976;112(1):1–35. doi: 10.1016/0006-8993(76)90331-0 947479

[58] Sotelo C, Arsenio-Nunes M. Development of Purkinje cells in absence of climbing fibers. Brain research. 1976;111(2):389–95. doi: 10.1016/0006-8993(76)90782-4 949602

[59] Kalinovsky A, Boukhtouche F, Blazeski R, Bornmann C, Suzuki N, Mason CA, et al. Development of axon-target specificity of ponto-cerebellar afferents. PLoS Biol. 2011;9(2):e1001013. doi: 10.1371/journal.pbio.1001013 21346800 PMC3035609

[60] Sillitoe RV. Mossy Fibers Terminate Directly Within Purkinje Cell Zones During Mouse Development. Cerebellum. 2016;15(1):14–7. doi: 10.1007/s12311-015-0712-6 26255945 PMC4729619

[61] Dorgans K, Demais V, Bailly Y, Poulain B, Isope P, Doussau F. Molecular and functional heterogeneity of cerebellar granule cell terminals expands temporal coding in molecular layer interneurons. bioRxiv. 2018:338152.

[62] Lordkipanidze T, Dunaevsky A. Purkinje cell dendrites grow in alignment with Bergmann glia. Glia. 2005;51(3):229–34. doi: 10.1002/glia.20200 15800897

[63] Uçar MC, Kamenev D, Sunadome K, Fachet D, Lallemend F, Adameyko I, et al. Theory of branching morphogenesis by local interactions and global guidance. Nature Communications. 2021;12(1):6830. doi: 10.1038/s41467-021-27135-5 34819507 PMC8613190

[64] Luczak A. Spatial embedding of neuronal trees modeled by diffusive growth. J Neurosci Methods. 2006;157(1):132–41. doi: 10.1016/j.jneumeth.2006.03.024 16690135

[65] Komuro H, Rakic P. Dynamics of granule cell migration: a confocal microscopic study in acute cerebellar slice preparations. Journal of Neuroscience. 1995;15(2):1110–20. doi: 10.1523/JNEUROSCI.15-02-01110.1995 7869087 PMC6577849

[66] Altman J. Experimental reorganization of the cerebellar cortex. V. Effects of early X-irradiation schedules that allow or prevent the acquisition of basket cells. Journal of Comparative Neurology. 1976;165(1):31–47. 1244360 10.1002/cne.901650104

