# Supplementary figures and images for: Models of Purkinje cell dendritic tree selection during early cerebellar development
Source: PLoS Comput Biol. 2023 Jul 24;19(7):e1011320. doi: 10.1371/journal.pcbi.1011320 (PMC10399850; doi:10.1371/journal.pcbi.1011320)

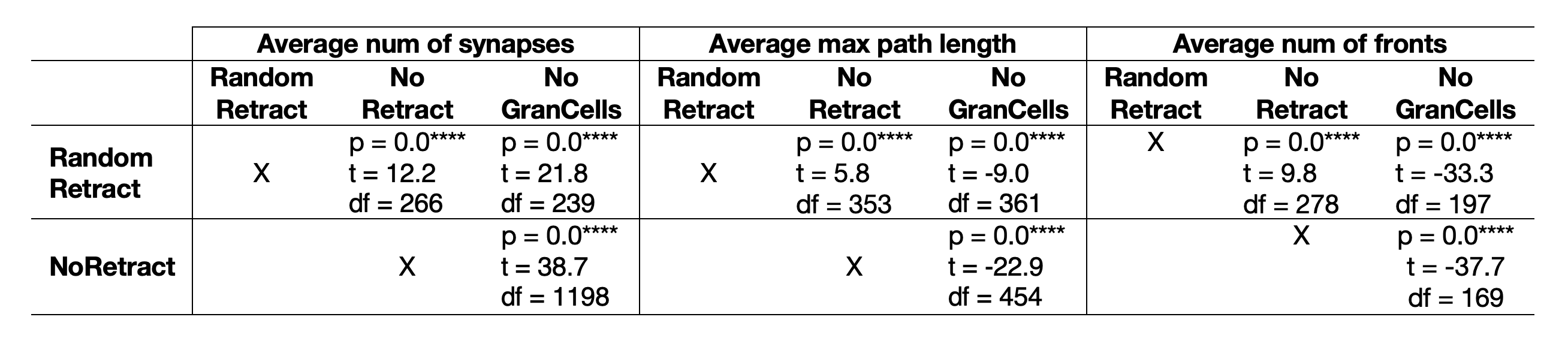

Supplement: S1 Table — (TIFF) [file pcbi.1011320.s001.tiff]

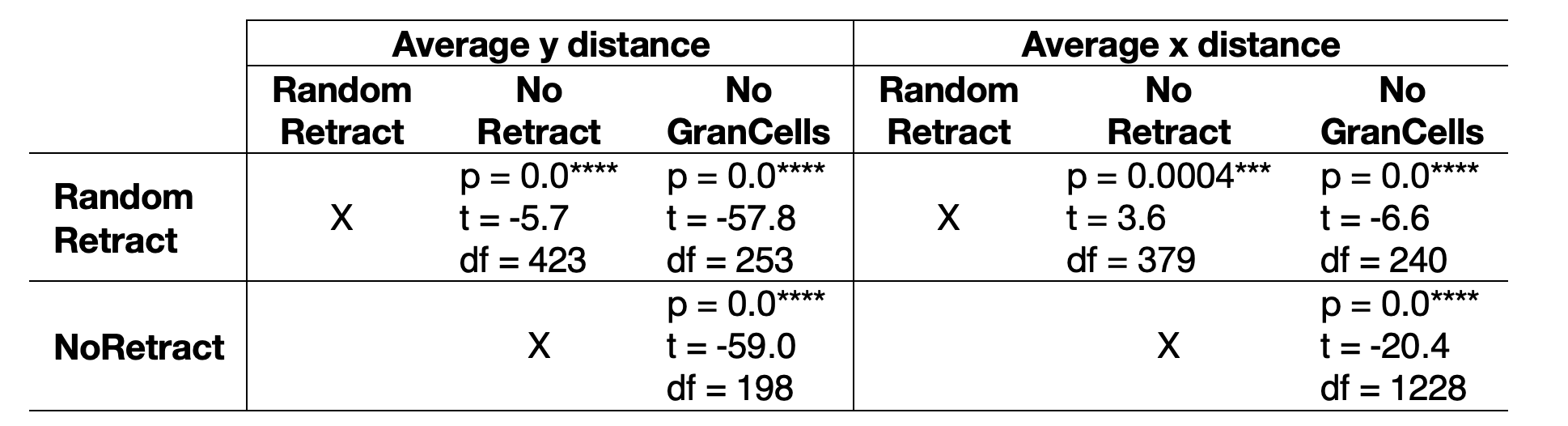

Supplement: S2 Table — (TIFF) [file pcbi.1011320.s002.tiff]

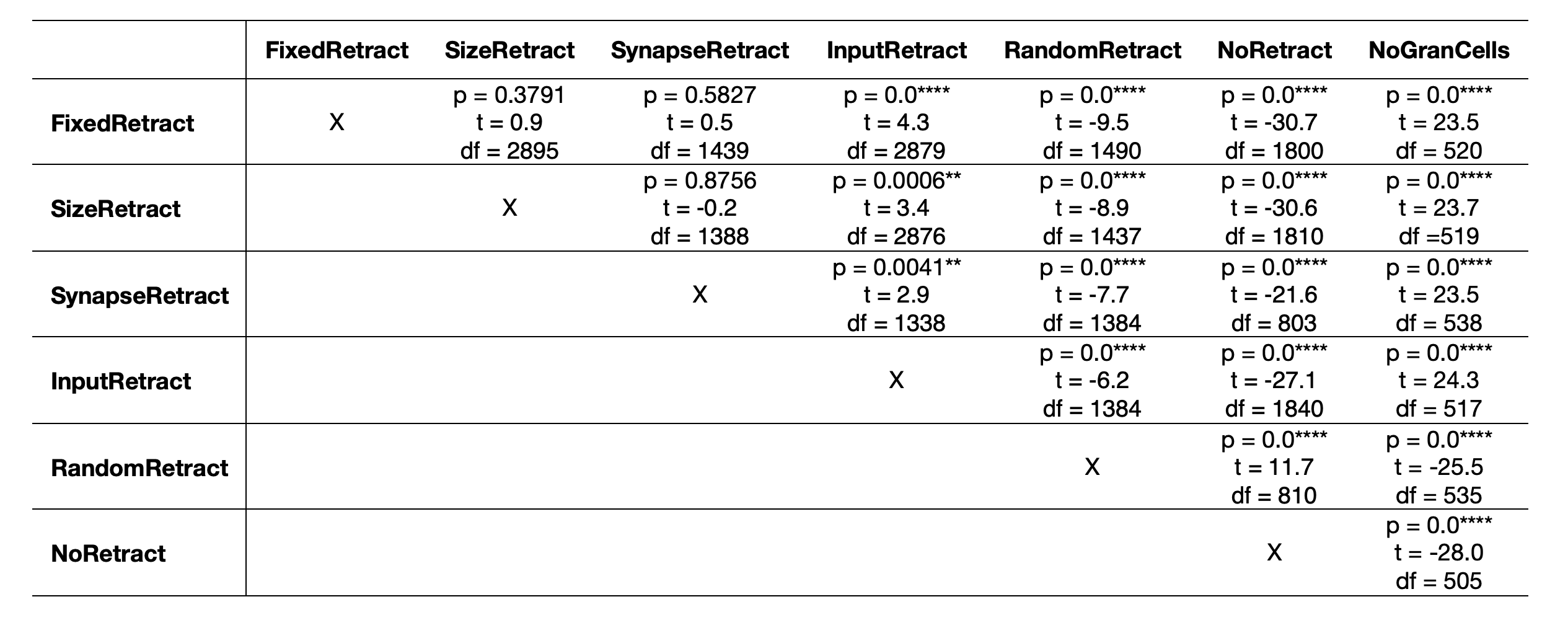

Supplement: S3 Table — (TIFF) [file pcbi.1011320.s003.tiff]

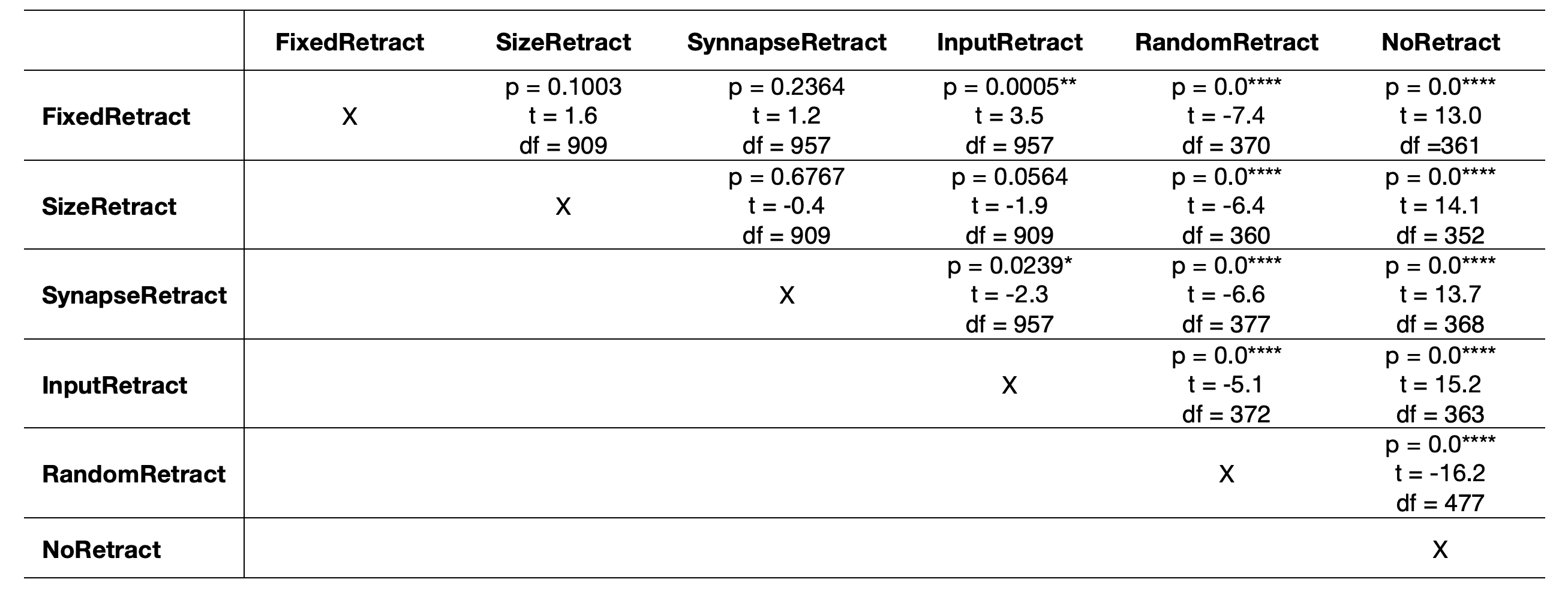

Supplement: S4 Table — (TIFF) [file pcbi.1011320.s004.tiff]

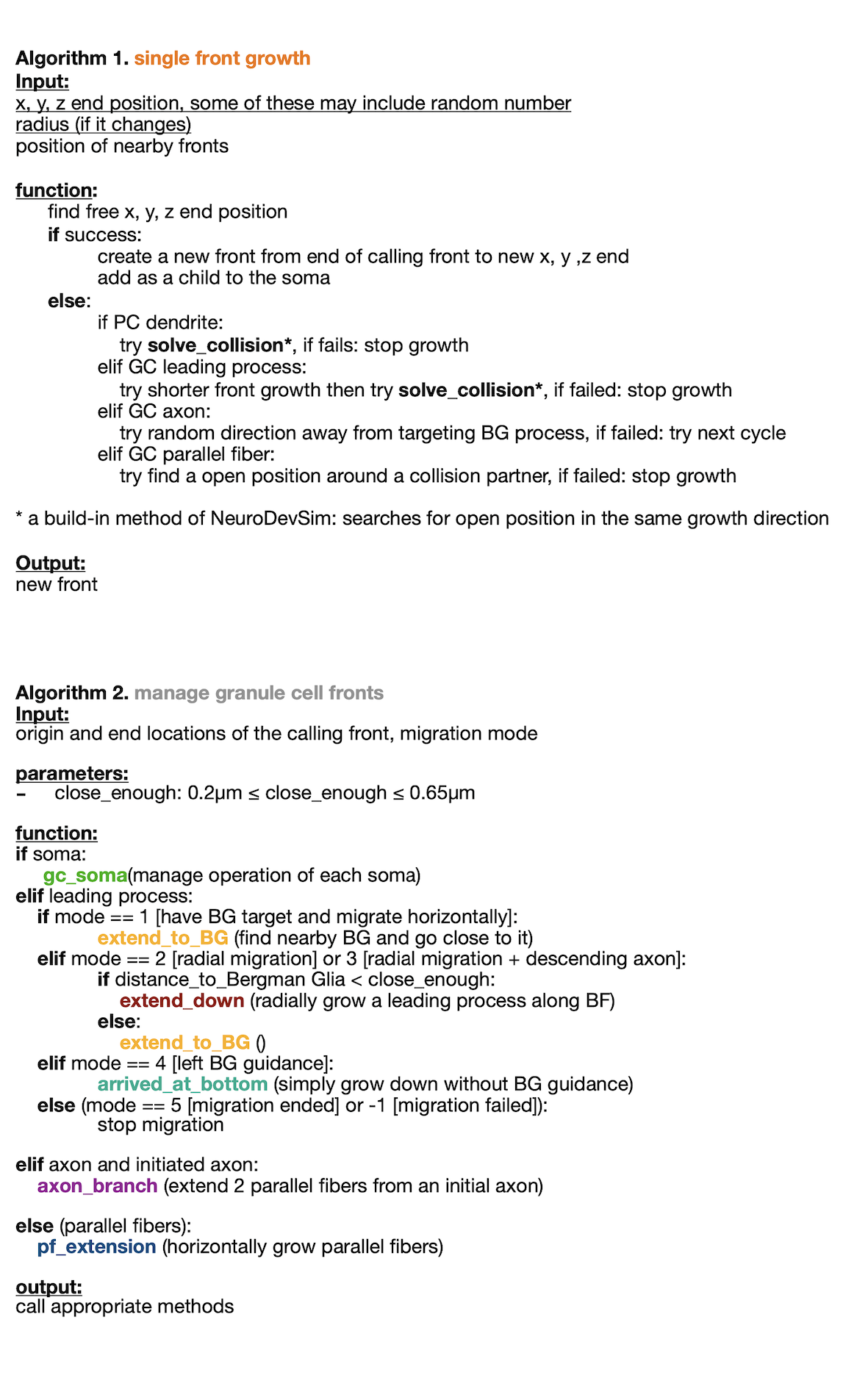

Supplement: S1 Fig — (TIFF) [file pcbi.1011320.s012.tiff]

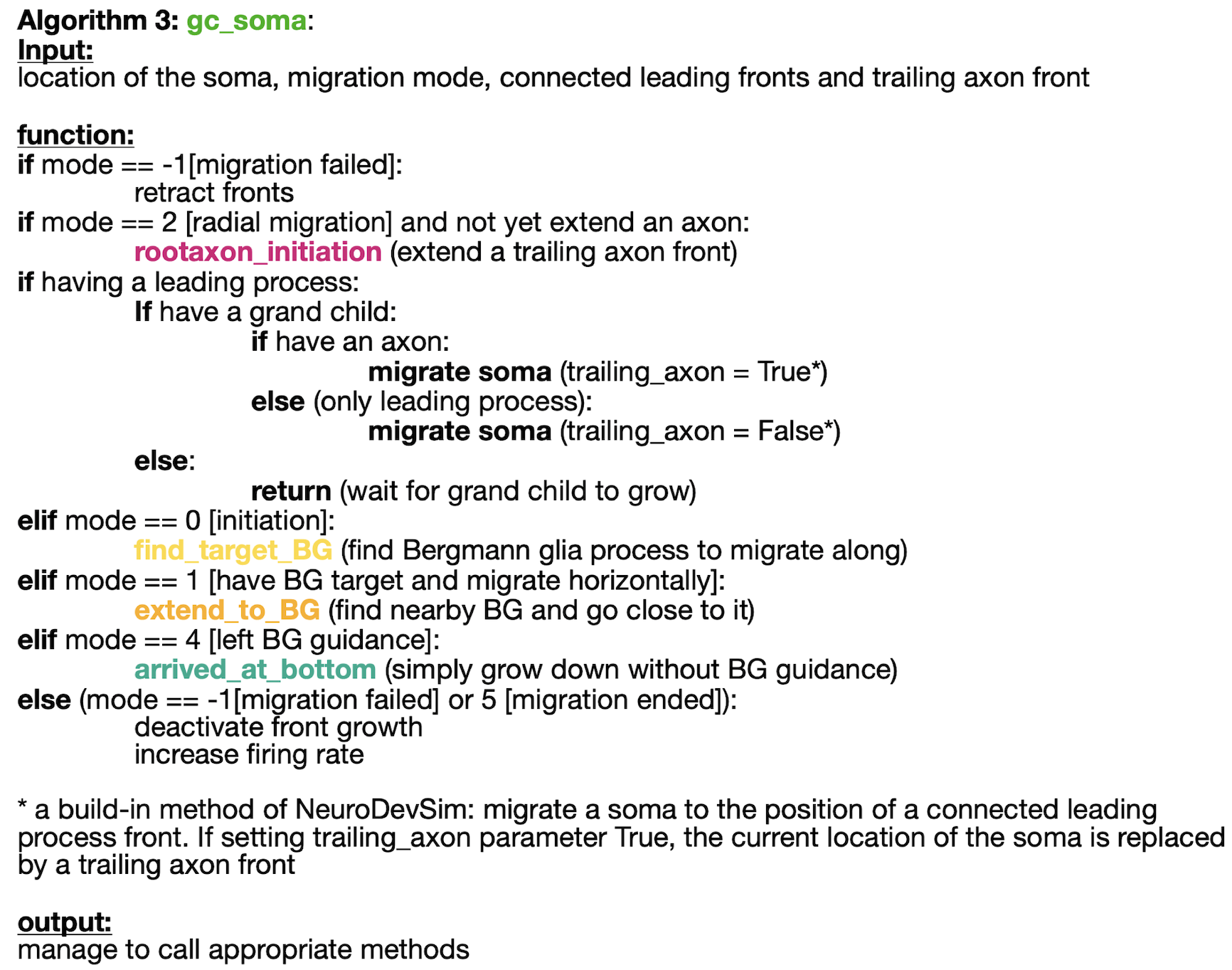

Supplement: S2 Fig — (TIFF) [file pcbi.1011320.s013.tiff]

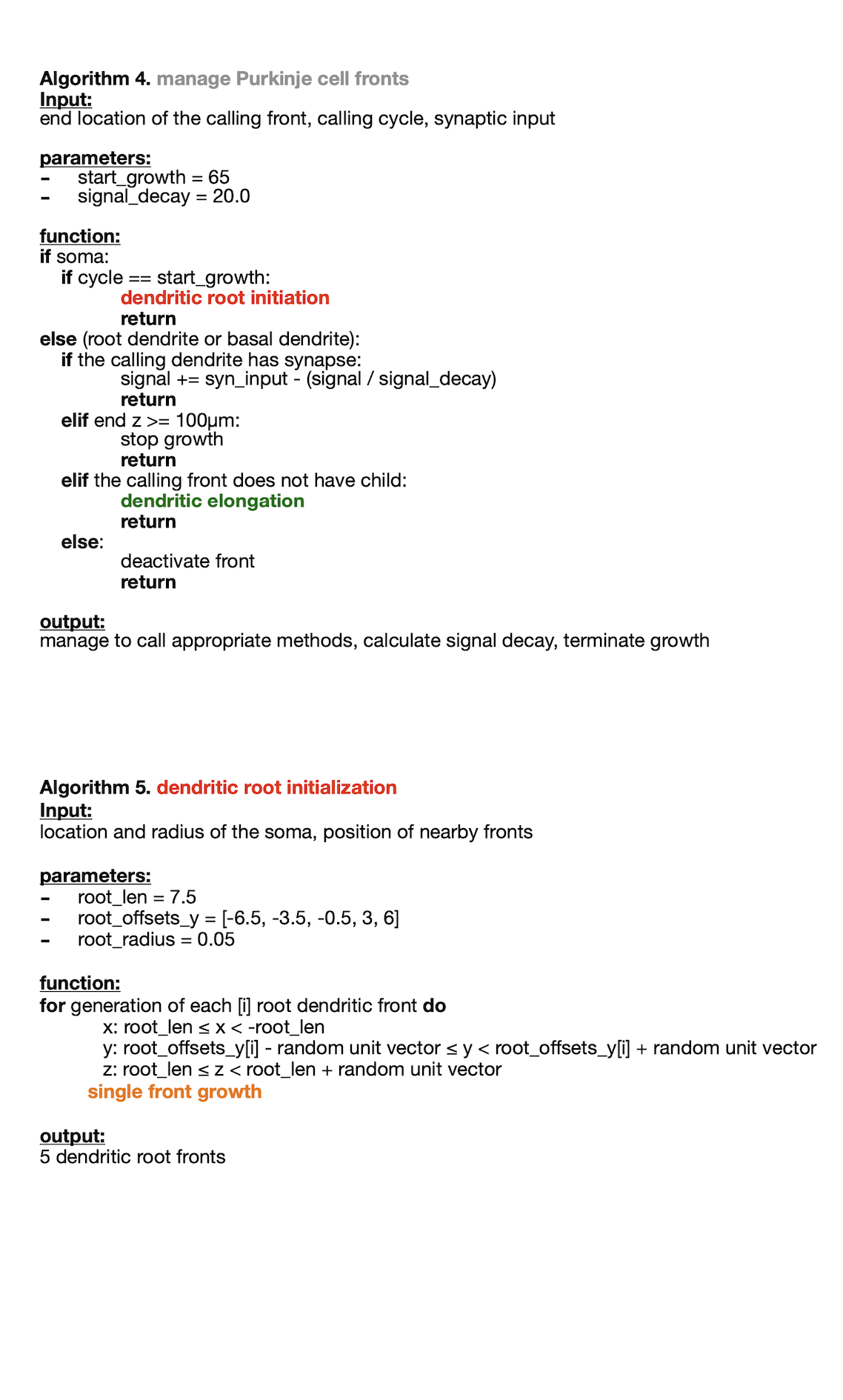

Supplement: S3 Fig — (TIFF) [file pcbi.1011320.s014.tiff]

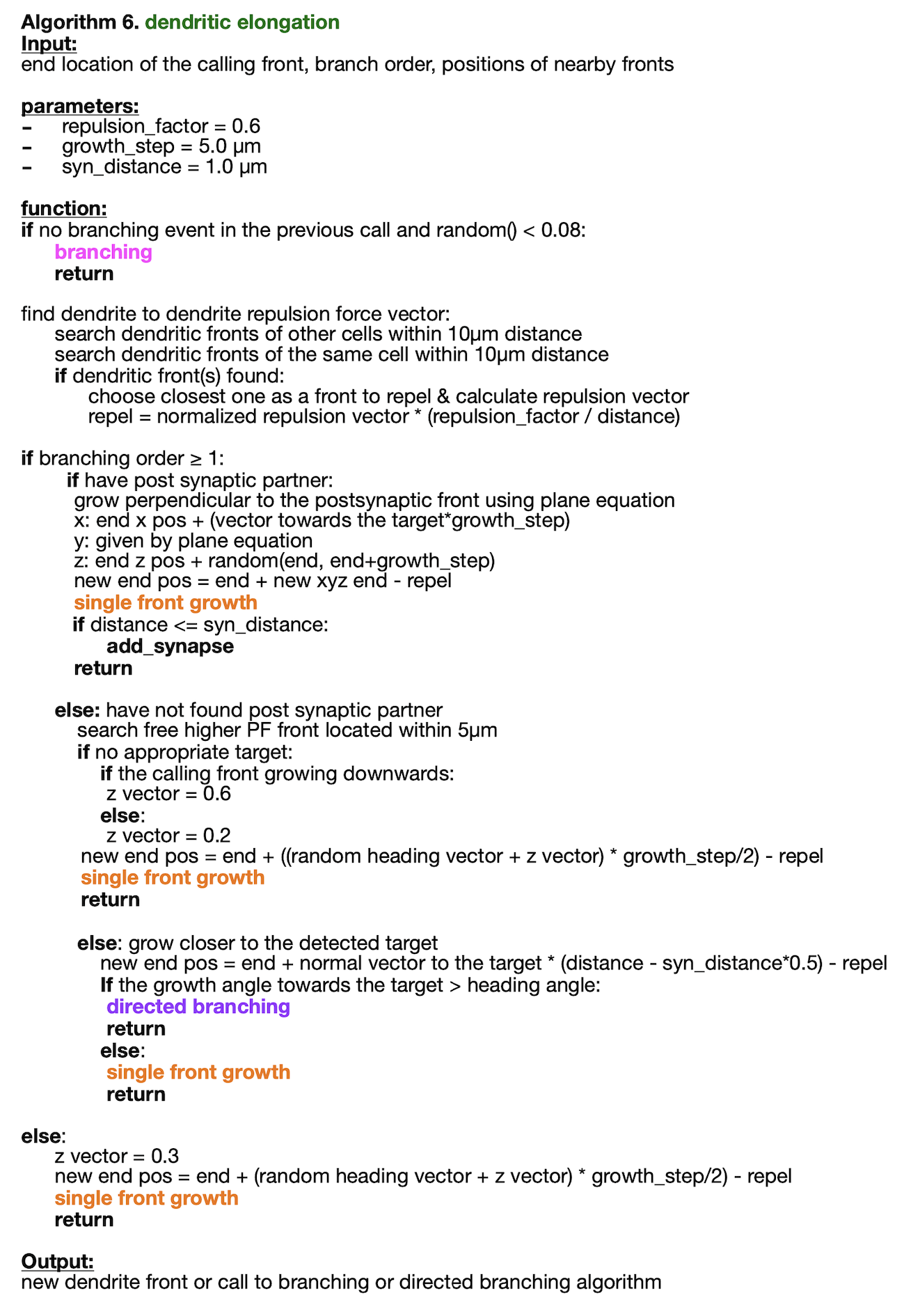

Supplement: S4 Fig — (TIFF) [file pcbi.1011320.s015.tiff]

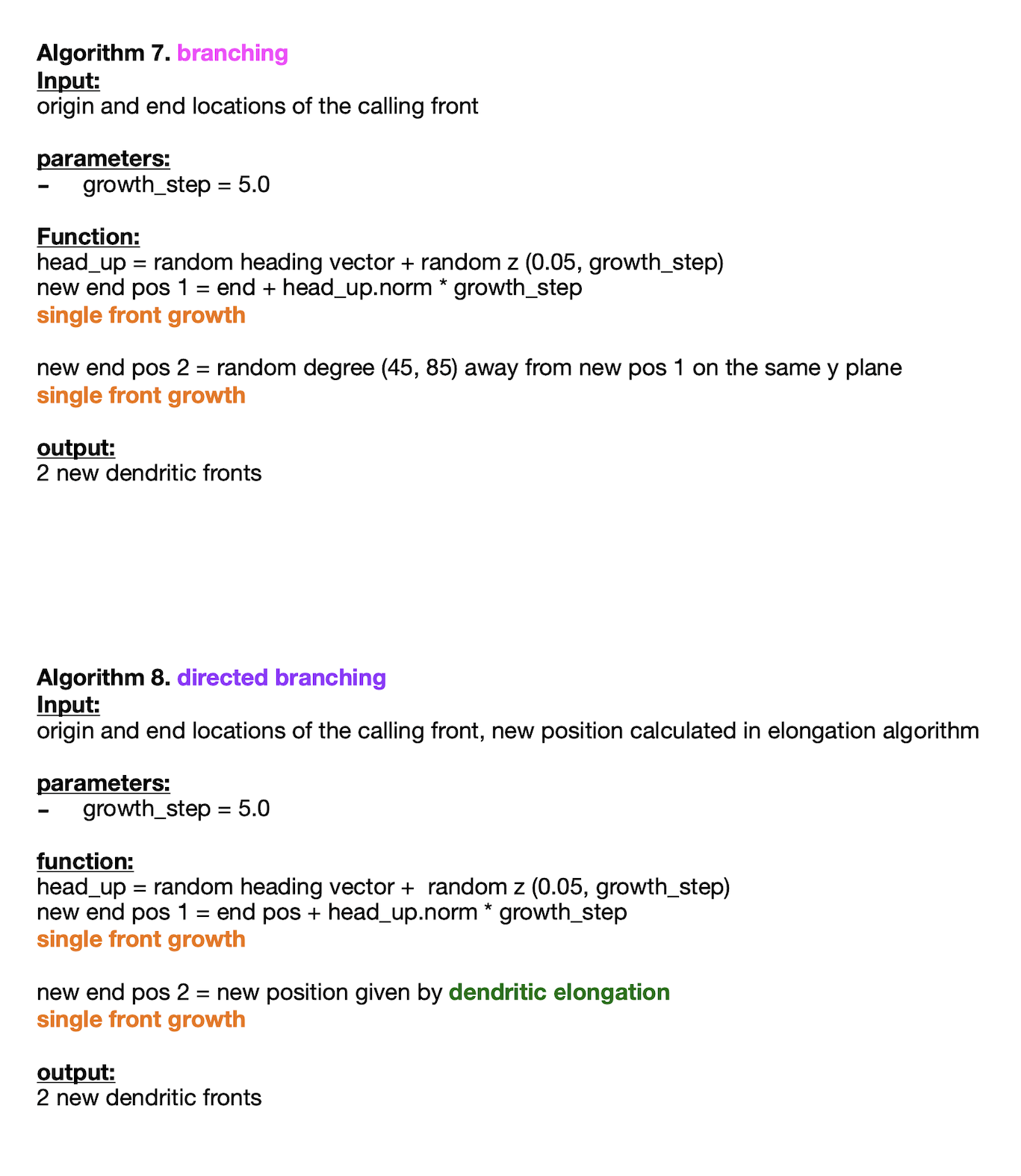

Supplement: S5 Fig — (TIFF) [file pcbi.1011320.s016.tiff]
